# Supplementary material for: Cumulative UV Exposure or a Modified SCINEXA™-Skin Aging Score Do Not Play a Substantial Role in Predicting the Risk of Developing Keratinocyte Cancers after Solid Organ Transplantation—A Case Control Study
Source: Cancers (Basel). 2023 Jan 30;15(3):864. doi: 10.3390/cancers15030864 (PMC9913211; doi:10.3390/cancers15030864)
Supplement: Supplementary file 1 [file cancers-15-00864-s001.zip › cancers-2144805-supplementary.pdf]

Supplementary Material

Cumulative UV Exposure or a Modified SCINEXA™-Skin Aging Score Do Not Play a Substantial Role in Predicting the Risk of Developing Keratinocyte Cancers after Solid Organ Transplantation—A Case Control Study

Liliane Borik-Heil, Georg Endler, Walther Parson, Andreas Zuckermann, Lisa Schnaller, Keziban Uyanik-Ünal, Peter Jaksch, Georg Böhmig, Daniel Cejka, Katharina Staufer, Elisabeth Hielle-Wittmann, Susanne Rasoul-Rockenschaub, Peter Wolf, Raute Sunder-Plassmann and Alexandra Geusau [1,*,‡]

Methods. Mitochondrial DNA Point Heteroplasmy
Results. Non melanoma skin cancers, Skin-aging score, UV-burden assessment, *MC1R* variants and their frequency in the whole study population, *MC1R* variants and their associations with the skin-aging scores and different UV-scenarios, MC1R variants and their associations with NMSC counts and phenotype, *MC1R* variants and their associations with NMSC entities

Table S1. Patient Characteristics (a) and Correlation of Melanin Index and Fitzpatrick Skin Type (I-IV) (b).
Table S2. Modified Skin Aging Scores Including the Intrinsic and Extrinsic Signs of Skin Aging in Cases and Controls.
Table S3. Recreational Activities in Total Weeks of Life in Cases and Controls
Table S4. Correlation between NMSC Count and Clinical/UV Associated Parameters.
Table S5. Reaction to Sun Exposure and Sunburns in Different Periods of Life.
Table S6. Characteristics of a Subset of Pairs Additionally Matched for Fitzpatrick Skin Type.
Table S7. The Number and Percentage of the Different Tumor Entities in Different Regions (a), Distribution of the Tumors in Different Localizations (b), Mean Number of Tumors per Patient and tumor entity (c).
Table S8. Correlation between NMSC Count and Age, Gender, Aging Score, Time to First NMSC, Number of AKs, and MC1R Risk Groups.
Table S9. Skin Aging Scores in a Subset of Cases and Controls Additionally Matched for Fitzpatrick Skin Type and in the Whole Study Population.
Table S10. Skin Aging Score Values in Association with Age and Gender.
Table S11. Skin Aging Scores in Association with Smoking History in the Whole Study Population, Subsets of FST-matched Cases/Controls Pairs and Cases with the Highest UV-burden.
Table S12. Mitochondrial DNA Point Heteroplasmy (PHP): Number of mtDNA PHP in UV-exposed Versus UV-unexposed Skin and in Cases Versus Controls
Table S13. Quantification of UV-Exposure in Different Scenarios and Different Subsets (cases versus controls).
Table S14. Correlations of the Skin Aging Scores with UV-exposure Scenarios in a Subset of 97 Cases/Control Pairs Matched for Fitzpatrick Skin Type.
Table S15. Correlations between Skin Aging Scores and NMSCs Located in Different Regions.
Table S16. Correlation of NMSC (localization) With UV Exposure.
Table S17. Comparison of The Skin Aging Scores and UV- Scenarios in Cases ≥5 NMSC: FST II Versus FST III Subsets.
Table S18. *MC1R* Variants and their Frequency in Cases Versus Controls (a), *MC1R* Haplotypes and Their Frequency in Cases and Controls (b), *MC1R* Diplotypes and Their Frequency in Cases and Controls (c).
Table S19. Association of *MC1R* Risk Groups with Skin Type, Hair and Eye Color in cases versus controls.
Table S20. *MC1R* Risk Groups in Different Subsets of Cases and Controls.
Table S21. Association of *MC1R* Risk Groups with Skin Pigmentation in Different Subgroups of OTR.

Table S22. Association of *MC1R* Risk Groups with Hair Color in Different Subgroups of OTR (≥5 and ≥10 NMSC vs matched controls).
Table S23. Association of *MC1R* Risk Groups with Eye Color in Different Subgroups of OTR (≥5 and ≥10 NMSC vs matched controls).
Table S24 Association of *MC1R* Risk Groups with Different UV Scenarios in Cases versus Controls (a, Part I and II), Association of *MC1R* Risk Groups with Different UV scenarios in Cases with ≥ 10 NMSC (n=44) versus Matched Controls (b, Part I and II).
Table S25. Association of *MC1R* Risk Groups with Hair Color in Different Subgroups of OTR (≥5 vs 1-4 and ≥10 vs 1-9 NMSC).
Table S26. Association of *MC1R* Risk Groups with Different NMSC Entities.

Figure S1. Hours of High UV-exposure (9am-3pm) in Mediterranean or Southern Regions in Cases versus Controls.

Supplemental material

Methods

Mitochondrial DNA Point Heteroplasmy

In a subset of 23 individuals, skin scales from the sun-exposed preauricular area and from non-UV-exposed buttocks were prepared for the evaluation of UVR-induced mitochondrial DNA (mtDNA) point heteroplasmy (PHP). Further details are in the supplement. Only superficial layers adjacent to the stratum corneum and lacking vasculature were used, as it was essential to avoid contamination with blood for the analysis of PHP in keratinocytes only. For massive parallel sequencing of the mitogenome in skin scales, total DNA was extracted from the skin scales using the QIAamp DNA Mini Kit (Qiagen, Hilden, Germany) on an EZ1 Advanced XL instrument (Qiagen) as described in Heidegger et al. (1), and mtDNA was specifically quantified using the real-time qPCR assay described in Xavier et al. (2). Massive parallel sequencing of the entire mitogenome was performed with the AmpliSeq-based Precision ID mtDNA Whole Genome Panel (ThermoFisher Scientific (TFS), Waltham, MA) using 3,000 – 5,000 copies mtDNA as input according to the protocol described in Strobl et al. (3) on an Ion S5 (TFS). Data were analyzed using Torrent Suite Software v5.2.1 (TFS) and reported relative to the rCRS (4) plus nucleotides 1–80 as an appendix after position 16,569. Bam/bai files were generated by the Torrent Suite software and variant calling was performed using the variant caller plug-in (v5.2.1.38). All mitogenome sequences were manually reviewed twice by two independent scientists using IGV (Integrative Genomics Viewer) (5). Point heteroplasmy (PHP) was determined by visual inspection of sequence raw data in IGV.

1. Heidegger, A.; Pisarek, A.; de la Puente, M.; Niederstatter, H.; Pospiech, E.; Wozniak, A.; Schury, N.; Unterlander, M.; Sidstedt, M.; Junker, K.; Ventayol Garcia, M.; Laurent, F.X.; Ulus, A.; Vannier, J.; Bastisch, I.; Hedman, J.; Sijen, T.; Branicki, W.; Xavier, C.; Parson, W.; Consortium, V. Development and inter-laboratory validation of the VISAGE enhanced tool for age estimation from semen using quantitative DNA methylation analysis. *Forensic Sci Int Genet* **2022**, *56*, 102596.
2. Xavier, C.; Eduardoff, M.; Strobl, C.; Parson, W. SD quants-Sensitive detection tetraplex-system for nuclear and mitochondrial DNA quantification and degradation inference. *Forensic Sci Int Genet* **2019**, *42*, 39-44.
3. Strobl, C.; Churchill Cihlar, J.; Lagace, R.; Wootton, S.; Roth, C.; Huber, N.; Schnaller, L.; Zimmermann, B.; Huber, G.; Lay Hong, S.; Moura-Neto, R.; Silva, R.; Alshamali, F.; Souto, L.; Anslinger, K.; Egyed, B.; Jankova-Ajanovska, R.; Casas-Vargas, A.; Usaquen, W.; Silva, D.; Barletta-Carrillo, C.; Tineo, D.H.; Vullo, C.; Wurzner, R.; Xavier, C.; Gusmao, L.; Niederstatter, H.; Bodner, M.; Budowle, B.; Parson, W. Evaluation of mitogenome sequence concordance, heteroplasmy detection, and haplogrouping in a worldwide lineage study using the Precision ID mtDNA Whole Genome Panel. *Forensic Sci Int Genet* **2019**, *42*, 244-251.
4. Andrews, R.M.; Kubacka, I.; Chinnery, P.F.; Lightowlers, R.N.; Turnbull, D.M.; Howell, N. Reanalysis and revision of the Cambridge reference sequence for human mitochondrial DNA. *Nat Genet* **1999**, *23*, 147.
5. Thorvaldsdottir, H.; Robinson, J.T.; Mesirov, J.P. Integrative Genomics Viewer (IGV): high-performance genomics data visualization and exploration. *Brief Bioinform* **2013**, *14*, 178-192.

Results

Non melanoma skin cancers

The anatomic localization of the different types of NMSC in the cases is shown in **Figure 1** and **Tables S7a-c).** The majority of NMSC (n=767, 47% of n=1633), and especially SCC (+Bow Ca without BD; 61% of n=620) were located in the head/neck region and 37% of NMSC in this region were BCC (of n=1633). One-third of the NMSC were located on the trunk, where most BCC (42% of n=733) were found, compared with 20% of the 620 SCC. In addition, 9% (n=139) of all NMSCs were

located in the 'lower extremity' region; as were 9% (n=65) of all BCCs and 5% (n=31) of all SCCs. For BDs, the majority (38%; n=106) were located in the head/neck region and 31% (n=86) on the trunk; the upper and lower extremities were similarly affected (16% (n=45) and 15% (n=43), respectively) (**Table S7b).** The ratio of SCC to BCC was 1.2 (SCC including BD):1 (BCC). Forty-one percent of cases versus 16% of NMSC-free controls had concomitant AKs in their dermatologic status ($p<0.001$) (**Table S2)**, this difference remained significant even in the subgroup of FST-matched pairs (data not shown). The number of NMSCs per patient in relation to specific tumor entities is shown in **Table S7c.**

Skin-aging score

The variables that contributed to this score included five signs of intrinsic and 15 signs of extrinsic skin aging. The frequency of occurrence or intensity of the signs of skin aging and the mean value for each variable are shown for cases and controls in **Table S2.** OTR who suffered from NMSCs had a significantly lower ISA score than the tumor-free controls (p=0.001). Controls had significantly higher signs of ESA, including pigmentary change, change in skin phototype, coarse wrinkles, dryness, comedones, and permanent erythema than cases, suggesting that none of these scores were associated with the presence of NMSCs.

When comparing cases (n=194) to controls (n=194), skin aging calculations showed significantly lower ISA, ESA and TSA scores in cases. When considering the entire study population (n=388) - regardless of KC development **-** the ESA score increased with age; patients ≥ 60 (n=310) versus < 60 (n=78) years at the time of evaluation. A higher TSA score was associated with male gender (p =0.043; males (n=298) versus females (n=90); **Table S10)**. All skin aging scores correlated with smoking history (smokers (n=243) versus non-smokers (n=145)) - demonstrating the reliability of our skin aging quantification. A higher ESA score correlated with the number of NMSCs in our study when considering cases with ≥5 NMSCs (n=87) versus cases with 1-4 NMSCs (n=107) or cases with ≥10 NMSCs (n=44) versus cases with 1-9 NMSCs (n=150).

In the subset of 97 pairs additionally matched for FST, all types of skin aging scores were significantly higher in controls, and slightly, but not significantly, higher or at least comparable in FSTII versus FSTIII subjects – except for ESA score (p=0.036). **Table S9. Tables S10 and S11** are discussed in the main manuscript. In this subset of 97 FST-matched pairs, all skin aging scores were significantly elevated in smokers (**Table S11**). It is important that cases and controls with a smoking history did not differ in any of the aging scores, regardless of their FST (**Table S11**). A subanalysis of FST-matched and AK-free case/control pairs showed no difference in the skin aging-scores between cases and controls, suggesting that additional factors contribute to KC development in this scenario (data not shown).

Assessment of UV exposure

The majority of study participants (n=370) were Austrians living near Vienna at an altitude <500m (**Table 1**), therefore residency related effects (accounting for latitude and altitude at place of residence) were the same for cases and controls (p=0.594). Of the few individuals who lived above 500 m, most were controls (11 controls vs. 7 cases). Outdoor occupation, e.g., construction worker or farmer, typically prior to organ transplantation, was not associated with the development of NMSC, as 37% of cases but 44% of controls reported past or present outdoor occupations, as reflected by a higher number of hours spent outdoors by occupation. Similarly, between May and August (higher UVR) control subjects spent more time outdoors on weekdays (recreational and occupational) and weekends (UV scenario I+II 'total time spent outdoors with unintentional sun exposure' in **Table 1),** generally exposing themselves to UVR unintentionally and thus, only partially (uncovered body parts). More cases reported skiing and water sports activities, but there was no significant difference when total weeks of life were calculated.

Detailed information on UV scenarios is described in the main document (**Table S13),** as are correlations with skin aging scores (**Table S14).** Mean hours of life in Mediterranean and Southern regions for cases and controls are described in detail in the main manuscript and are shown in **Figure S1**).

Table S15: As indicated in the main document, SCC (total, head/neck and trunk region), BCC (total, head/neck region, upper extremities) correlated significantly with all types of skin aging scores (range $p<0.001$ to p=0.025).

UV exposure, measured by hours of life, was not associated with tumor burden when comparing cases with ≥10 NMSCs to those with less than 10 tumors; this was also independent of FST. However, beach vacations in Central Europe and Southern geographic regions were associated with higher tumor burden. This difference was not apparent when cases with less than five and ≥5 NMSCs were compared.

In addition, we observed a significant correlation of the ESA and TSA scores with the number of SCCs on the upper extremities (*p*=0.007 and 0.036, respectively), while for the lower extremities only the TSA score was significant (*p*=0.041). There was also a significant correlation for BCC in the 'head/neck', 'trunk' and 'upper extremities' locations with all variations of the skin aging scores, except for BCC on the trunk, which did not correlate with the ESA score.
Table S16 and Table S17 are described in detail in the main document**.**

***MC1R* variants and their frequency in the entire study population**

The *MC1R* genotype was available for each patient included in this study (n=388); **Table S18a** summarizes detailed information on the genetic variability of *MC1R* in our study populatione, i.e., *MC1R* variants (n=31), haplotypes (n=19) and diplotypes (n=43; **Tables S18a-c**). *MC1R* is highly polymorphic in Europeans. Several common variants including rs1805006 (c.252C>A, p.Asp84Glu), rs11547464 (c.425G>A, p.Arg142His), rs1805007 (c.451C>T, p.Arg151Cys), rs1110400 (c.464T>C, p.Ile155Thr), rs1805008 (c.478C>T, p.Arg160Trp) and rs1805009 (c.880G>C, p.Asp294His) associated with a 'red hair color phenotype' ('R' alleles; red hair, fair skin, freckles, sun sensitivity - solar lentigines and low tanning tendency). In addition, variants with a weaker association with red hair color phenotype (including rs1805005 (c.178G>T, p.Val60Leu), rs2228479 (c.274G>A, p.Val92Met), rs885479 (c.488G>A, p.Arg163Gln) were defined as "r" alleles.[13-15] Functional analysis of the R and r variants suggests impaired activation of the cAMP pathway, leading to a quantitative shift in eumelanin to phaeomelanin synthesis [16] (**Tables S18a-c**).

Participants who did not carry an *MC1R* variant (n=91) and individuals who carried variants that were unlikely to affect receptor function (based on published data or as predicted by bioinformatic mutation analysis tools; n=40) were considered as *MC1R* 0/0 risk group (n=131). Together with heterozygous carriers of *MC1R* r variants (0/r; n=126; reduced receptor function), these individuals were assigned to the *MC1R* low-risk group (0/0 plus 0/r; n=257). Medium genetic risk for *MC1R* dysfunction (n=119) was defined by the presence of either heterozygous R (0/R; n=69; loss of function), compound heterozygous Rr (n=26), or homo- or compound heterozygous rr (n=24) variants. Individuals assigned high *MC1R* risk carried homozygous or compound heterozygous RR variants (n=12).

The different *MC1R* risk types were associated with pigmentation phenotypes, i.e., individuals with FST I predominantly (89%) carried R/R high-risk variants, whereas the majority of participants with FST II and III carried no (0/0; 30% and 38%, respectively) or low-risk 0/r (32% and 34%, respectively) variants (n.s) (**Table S21**). Associations of *MC1R* variants with hair color are also described in the main manuscript.
Other associations of the *MC1R* risk types with phenotypes or development of NMSC or tumor number are shown in **Tables S21-24**. We found the highest percentage of low-risk *MC1R* variants in individuals with dark brown and black hair (74% and 82%, respectively; **Table S22**). Considering an association of eye color and *MC1R* with the development of NMSC independent of FST, significant differences in *MC1R* genotypes (low versus medium+high risk variants) were observed only in blue-eyed individuals (*p*=0.024); cases were more likely (43%) to carry medium plus high-risk variants than controls (25%; **Table S23**). This difference remained significant when cases with high tumor counts (≥5 or ≥10) were compared with matched controls (*p*=0.007 and *p*=0.028, respectively; **Table S25**). Cases with blue eyes and a tumor number of ≥5 NMSCs were not significantly different from those with lower tumor numbers (1-4 NMSC; *p*=0.169), while those with even higher tumor burden (≥10 NMSC) reached borderline significance compared to those with less than 10 NMSCs (*p*=0.051; **Table S25)**. Individuals with green or brown eyes and a tumor count of less than 10 NMSCs were significantly more likely to carry low-risk variants than individuals with a tumor count of ≥10 NMSCs (*p*= 0.040, *p*=0.033, respectively; **Table S25**).

MC1R risk groups in cases and controls - considering the entire study population - and self-reported UV exposure did not correlate **Table S24.**

In high-risk variant carriers, the ESA and TSA score values increased in cases but not in controls; in the latter, the ISA score increased and remained slightly higher than in cases. When cases and controls were compared within the *MC1R* medium- or high-risk groups, no significant difference in skin aging scores was found, except for a correlation between higher ESA score values and high-risk variants in cases**.** However,the number of individuals, was very small (n=12; *p*=0.02). Coversely, controls at low risk for MC1R dysfunction had significantly higher scores in all types of skin aging scores than cases (*p*=0.003 ISA score; *p*=0.046 ESA score; *p*=0.013 TSA score).

MC1R variants and their associations with different UV scenarios are shown in **Table S24**, and MC1R variants and their associations with NMSC count and phenotype are shown in **Table S24, S25**.

Higher NMSC counts clearly correlated with the medium+high-risk *MC1R* group, when cases with a history of ≥5 or ≥10 NMSCs were compared with either those with 1-4 (p=0.004) or 1-9 (p=0.001) NMSCs (**Figure 4, Table S22),** or with their matched controls (p=0.008 and p=0.019, respectively; **Figure 4, Table S22**). The significance level increased when comparing FSTII cases with ≥5 NMSCs to their matched controls (p=0.008) (**Table S21**); when analyzing cases with ≥10 NMSCs, no significance but a trend was observed for these calculations (p=0.088), most likely due to the small number of individuals in this subgroup (see also **Table S21**). The majority of individuals with FSTI (n=9) carried *MC1R* high-risk variants (R/R; n=8), but the number of R/R carriers was too small for meaningful statistical conclusions. However, more controls (n=5) with FSTI were in the *MC1R* high-risk group.

The *MC1R* gene is highly polymorphic in the Caucasian population. Several common variants are associated with the 'red hair color/fair skin type phenotype' ('R' alleles), variants with a weaker association with the red hair color phenotype were defined as "r" alleles. Functional analyses of r and R variants suggest impaired activation of the cAMP pathway leading to a quantitative shift ineumelanin to less protective phaeomelanin synthesis [16]. Carriers of the phenotypic *MC1R* 'R' and/or 'r' variants were found to be at higher risk for UV-induced skin damage and, consequently, melanoma and NMSC. Tagliabue et al. reported that individuals carrying at least one *MC1R* 'R' or 'r' variant have an increased overall risk of developing NMSC, particularly BCC and SCC. In our population, the distribution of 0/r was comparable between cases and controls. No particular *MC1R* variant was significantly associated with the development of skin tumor, with the exception of the relatively rare c.464T>C, p.Ile155Thr**,** one of the classical R variants, which was detected exclusively in 6 cases.

***MC1R* variants and their associations with NMSC entities**

The distribution of the different *MC1R* genotypes in patients preferentially affected by particular tumor types revealed the following: Patients who developed a single tumor entity (cSCC, BCC, or BD) exclusively, carried predominantly low-risk variants (71%, 74%, and 80%, respectively). In patients with the full spectrum of NMSC, the distribution of low and medium+high-risk *MC1R* types was equal (**Table S26**).

Multivariate regression analysis (Figure 5)

A forest plot showing odds ratios and 95% confidence intervals for: *MC1R* risk groups, eye colors and AKs, adjusted for all variables collected, including FST, freckles, moles, skin aging scores, smoking habits, primary residence (altitude), and extent of UV exposure (time spent outdoors with intentional and unintentional sun exposure, outdoor occupation). Odds ratios (diamonds) and 95% confidence intervals (solid horizontal lines) are shown on the x-axis. The dashed vertical line indicates an OR value of 1 (no effect). Factors identified in multivariable models that were significantly associated with the development of NMSC included blue eyes (OR: 3.6; 95% CI: 2.02-6.28; p<0.001), green eyes (OR: 4.1; 95% CI: 1.88-8.81; p<0.001), intermediate+high risk *MC1R* variants (OR: 1.9; 95% CI: 1.13-3.08; p=0.015) and AKs (OR: 7.5; 95% CI: 4.08-13.68; p<0.001).

References

1. Fitzpatrick TB. The validity and practicality of sun-reactive skin types I through VI. *Arch Dermatol*. 1988;124(6):869-71.

2. Dwyer T, Muller HK, Blizzard Let al. The use of spectrophotometry to estimate melanin density in Caucasians. *Cancer Epidemiol Biomarkers Prev*. 1998;7(3):203-6.

3. Eilers S, Bach DQ, Gaber Ret al. Accuracy of self-report in assessing Fitzpatrick skin phototypes I through VI. *JAMA Dermatol*. 2013;149(11):1289-94.

4. Sitek A, Rosset I, Zadzinska Eet al. Skin color parameters and Fitzpatrick phototypes in estimating the risk of skin cancer: A case-control study in the Polish population. *J Am Acad Dermatol*. 2016;74(4):716-23.

5. Buranasirin P, Pongpirul K, Meephansan J. Development of a Global Subjective Skin Aging Assessment score from the perspective of dermatologists. *BMC Res Notes*. 2019;12(1):364.

6. Fuks KB, Huls A, Sugiri Det al. Tropospheric ozone and skin aging: Results from two German cohort studies. *Environ Int*. 2019;124(139-44.

7. Vierkotter A, Ranft U, Kramer Uet al. The SCINEXA: a novel, validated score to simultaneously assess and differentiate between intrinsic and extrinsic skin ageing. *J Dermatol Sci*. 2009;53(3):207-11.

8. Heidegger A, Pisarek A, de la Puente Met al. Development and inter-laboratory validation of the VISAGE enhanced tool for age estimation from semen using quantitative DNA methylation analysis. *Forensic Sci Int Genet*. 2022;56(102596.

9. Xavier C, Eduardoff M, Strobl Cet al. SD quants-Sensitive detection tetraplex-system for nuclear and mitochondrial DNA quantification and degradation inference. *Forensic Sci Int Genet*. 2019;42(39-44.

10. Strobl C, Churchill Cihlar J, Lagace Ret al. Evaluation of mitogenome sequence concordance, heteroplasmy detection, and haplogrouping in a worldwide lineage study using the Precision ID mtDNA Whole Genome Panel. *Forensic Sci Int Genet*. 2019;42(244-51.

11. Andrews RM, Kubacka I, Chinnery PFet al. Reanalysis and revision of the Cambridge reference sequence for human mitochondrial DNA. *Nat Genet*. 1999;23(2):147.

12. Thorvaldsdottir H, Robinson JT, Mesirov JP. Integrative Genomics Viewer (IGV): high-performance genomics data visualization and exploration. *Brief Bioinform*. 2013;14(2):178-92.

13. Garcia-Borron JC, Olivares C. Melanocortin 1 receptor and skin pathophysiology: beyond colour, much more than meets the eye. *Exp Dermatol*. 2014;23(6):387-8.

14. Raimondi S, Sera F, Gandini Set al. MC1R variants, melanoma and red hair color phenotype: a meta-analysis. *Int J Cancer*. 2008;122(12):2753-60.

15. Tagliabue E, Gandini S, Garcia-Borron JCet al. Association of Melanocortin-1 Receptor Variants with Pigmentary Traits in Humans: A Pooled Analysis from the M-Skip Project. *J Invest Dermatol*. 2016;136(9):1914-7.

16. Duffy DL, Box NF, Chen Wet al. Interactive effects of MC1R and OCA2 on melanoma risk phenotypes. *Hum Mol Genet*. 2004;13(4):447-61.

Supplementary Tables

Table S1a. Patient characteristics

	Total n=388	Cases n=194	Controls n=194	*p* value
**Gender** *(n (%))*				
Male	298 (77)	149 (77)	149 (77)	
Female	90 (23)	45 (23)	45 (23)	
**Type of organ transplant recipient** *(n*				
HTR	164 (42)	82 (42)	82 (42)	
KTR	148 (38)	74 (38)	74 (38)	
LTR	38 (10)	19 (10)	19 (10)	
LuTR	38 (10)	19 (10)	19 (10)	
**Age at visit,** years *(mean ± SD)*	68.2 ± 9.1	68.4 ± 9.3	68.0 ± 9.0	
**Age at TX,** years *(mean ± SD)*	54.7 ± 12.2	54.9 ± 12.4	54.0 ± 12.5	
**Post-TX period,** years *(mean ± SD)*	13.5 ± 7.8	13.5 ± 7.9	13.5 ± 7.8	
**Time to 1st post-TX NMSC,** years	7.9 ± 7.1	7.9 ± 7.1	0	
**IS-regimen** *(n (%))*				
Steroids Yes	193 (50)	93 (48)	100 (52)	
No	195 (50)	101 (52)	94 (49)	
mTOR inhibitors Yes	90 (23)	48 (25)	42 (22)	
No	298 (77)	146 (75)	152 (78)	
Antimetabolites Yes	281 (72)	135 (70)	146 (75)	
No	107 (28)	59 (30)	48 (25)	
Calcineurin inhibitors Yes	307 (79)	155 (80)	152 (78)	
No	81 (21)	39 (20)	42 (22)	
Azathioprine Yes	24 (6)	11 (6)	13 (7)	
No	364 (94)	183 (94)	181 (93)	
Belatacept Yes	7 (2)	2 (1)	5 (3)	
No	381 (98)	192 (99)	189 (97)	
**Skin type** *(n (%))*				
I	9 (2)	4 (2)	5 (3)	**<0.001**
II	177 (46)	107 (55)	70 (36)	
III	199 (51)	83 (43)	116 (60)	
IV	3 (1)	0	3 (2)	
**Reaction to sun exposure** *(n (%))*				
Rather red	185 (49)	110 (57)	75 (39)	**<0.001**
Rather brown	203 (52)	84 (43)	119 (61)	
**Hair color** *(n (%))*				
Red	7 (2)	2 (2)	5 (3)	**<0.001**
Blond	116 (30)	73 (38)	43 (22)	
Light brown	100 (26)	60 (31)	40 (21)	
Dark brown	137 (35)	53 (27)	84 (43)	
Black	28 (7)	6 (3)	22 (11)	
**Eye color** *(n (%))*				
Blue	148 (38)	92 (47)	56 (29)	**<0.001**
Grey	56 (14)	24 (12)	32 (17)	
Green	51 (13)	32 (17)	19 (10)	
Brown	133 (34)	46 (24)	87 (45)	
**Childhood freckling** *(n (%))*				
Yes	86 (22)	53 (27)	33 (17)	**0.020**
No	302 (78)	141 (73)	161 (83)	
**Patients** *(n (%))* **presenting with**				
1-9	148 (38)	77 (40)	71 (37)	0.958
10-29	118 (30)	58 (30)	60 (31)	
30-100	78 (20)	39 (20)	39 (20)	
>100	22 (6)	10 (5)	12 (6)	
No moles	22 (6)	10 (5)	12 (6)	
**number of seborrheic warts**				
1-9	179 (46)	95 (49)	84 (43)	0.200
10-30	76 (20)	41 (21)	35 (18)	
No seborrhoic warts	133 (34)	58 (30)	75 (39)	
**bald head** (Norwood scale, males	298	149	149	
Mild - moderate (I - V)	172 (58)	77 (52)	95 (64)	**0.020**
Severe (VI, VII)	88 (30)	55 (37)	33 (22)	
No baldness	36 (12)	17 (11)	19 (13)	
**Smoking history** *(n (%))*				
Yes, currently	27 (7)	10 (5)	17 (9)	0.056
Yes, in the past	216 (56)	101 (52)	115 (59)	
No, never	145 (37)	83 (43)	62 (32)	

$p<0.05$ is considered significant, indicated in bold. HTR=heart transplant recipient, KTR=kidney transplant recipient, LTR=liver transplant recipient, LuTR=lung transplant recipient, TX=transplantation, NMSC=non-melanoma skin cancer, SD=standard deviation, IS=immunosuppression.

Table S1b. Correlation of melanin index and Fitzpatrick skin type (I-IV)

	**Fitzpatrick skin type**					
	**I (n=9)**	**II (n=177)**	**III (n=199)**	**IV (n=3)**	r_s	***p* value**
**MI Buttocks** *(mean ± SD)*	23.4 ± 1.8	26.5 ± 2.4	31.8 ± 2.8	38.7 ± 1.5	0.817	**<0.001**
**MI Forehead** *(mean ± SD)*	33.6 ± 3.6	33.3 ± 3.5	34.6 ± 4.2	38.7 ± 3.0	0.172	**0.001**
**MI Hand** *(mean ± SD)*	34.5 ± 3.7	36.8 ± 5.4	38.3 ± 7.0	38.2 ± 9.9	0.121	**0.017**

$p<0.05$ is considered significant, indicated in bold. MI=Melanin index, SD=standard deviation, r_s=Spearman correlation coefficient.

Table S2. Modified skin aging scores including the intrinsic and extrinsic signs of skin aging in cases and controls

				Number of OTR affected *n (%)*				Score *mean ± SD*			
	**Skin aging signs**	**Localization**	**Ordinal scale**	**Total**	**Cases**	**Controls**	***p* value**	**Total**	**Cases**	**Controls**	***p* value**
**Intrinsic skin aging signs**	Lax appearance	Face and neck	0-3	362 (93)	179 (93)	183 (94)	0.417	1.67 ± 0.89	1.52 ± 0.86	1.82 ± 0.90	**0.001**
	Uneven pigmentation	Face	0/3	156 (40)	73 (38)	83 (43)	0.300	1.21 ± 1.47	1.13 ± 1.46	1.28 ± 1.49	0.302
	Fine wrinkles	Around eye-lid, cheeks	0-3	360 (93)	177 (91)	183 (94)	0.239	1.66 ± 0.92	1.60 ± 0.91	1.73 ± 0.92	0.166
	Benign skin tumors	Head and neck	0-3	190 (49)	80 (41)	110 (57)	**0.002**	0.64 ± 0.77	0.56 ± 0.77	0.72 ± 0.75	**0.034**
	Reduced fat tissue	Cheeks	0-3	285 (74)	137 (71)	148 (76)	0.206	1.18 ± 0.91	1.10 ± 0.90	1.26 ± 0.93	0.076
**ISA score**								6.36 ± 2.77	5.90 ± 2.78	6.81 ±2.68	**0.001**
**Extrinsic skin aging signs**	Sunburn freckles	Shoulders	0-3	221 (57)	112 (58)	109 (56)	0.758	0.98 ± 1.05	1.00 ± 1.07	0.92 ± 1.02	0.308
	Lentigines solaris	Forehead, cheeks, dorsum of forearms and hands (mean values)	0-3	365 (94)	186 (96)	179 (92)	0.132	1.53 ± 0.84	1.62 ± 0.84	1.45 ± 0.84	**0.047**
	Pigment change	Face vs less UV exposed skin	0-3	245 (63)	103 (53)	142 (73)	**<0.001**	0.99 ± 0.92	0.79 ± 0.85	1.19 ± 0.95	**<0.001**
	Change of skin phototype		0-3	75 (19)	26 (13)	49 (25)	**0.002**	0.27 ± 0.58	0.16 ± 0.42	0.38 ± 0.70	**<0.001**
	Yellowness	Face	0-3	31 (8)	12 (6)	19 (10)	0.190	0.01 ± 0.35	0.08 ± 0.32	0.12 ± 0.38	0.251
	Pseudoscars	Face	0-3	12 (3)	6 (3)	6 (3)	1.000	0.05 ± 0.29	0.04 ± 0.27	0.05 ± 0.32	0.729
	Coarse wrinkles	Forehead, crow´s feet, infraorbital, upper lip region, nasolabial (mean values)	0-3	386 (99)	192 (99)	194 (100)	0.156	2.02 ± 0.62	1.94 ± 0.63	2.09 ± 0.61	**0.022**
	Elastosis cutis	Face	0-3	298 (77)	146 (75)	152 (78)	0.470	1.37 ± 1	1.35 ± 1.02	1.39 ± 0.99	0.686
	Cutis rhomboidalis nuchae	Neck	0/3	113 (29)	61 (31)	52 (27)	0.315	0.87 ± 1.36	0.94 ± 1.39	0.80 ± 1.33	0.333
	M. Favre Racouchot	Face	0/3	31 (8)	14 (7)	17 (9)	0.574	0.24 ± 0.81	0.22 ± 0.78	0.26 ± 0.85	0.575
	Dryness	Face, back of forearm	0-3	102 (26)	30 (16)	72 (37)	**<0.001**	0.34 ± 0.63	0.20 ± 0.51	0.49 ± 0.69	**<0.001**
	Comedones	Periorbital	0-3	62 (16)	21 (11)	41 (21)	**0.009**	0.24 ± 0.70	0.14 ± 0.45	0.33 ± 0.86	**0.007**
	Telangiectasis	Cheeks, nose	0-3	270 (70)	134 (69)	136 (70)	0.737	1.03 ± 0.89	1.02 ± 0.89	1.04 ± 0.89	0.817
	Permanent erythema	Neck, decollete	0-3	296 (76)	135 (70)	161 (83)	**0.002**	1.42 ± 1.05	1.22 ± 1.03	1.62 ± 1.03	**<0.001**
	Actinic keratosis	Whole skin	0/3	110 (28)	80 (41)	30 (16)	**<0.001**	0.85 ± 1.35	1.24 ± 1.48	0.46 ± 1.09	**<0.001**
**ESA score**								12.26 ± 5.01	11.96 ± 5.02	12.56 ± 4.99	0.236
**TSA score**								18.63 ± 6.98	17.90 ± 7.00	19.36 ± 6.90	**0.040**

p<0.05 is considered significant, indicated in bold. OTR=organ transplant recipients, SD=standard deviation, ISA score=intrinsic skin aging score, ESA score=extrinsic skin aging score, TSA score=total skin aging score.

Table S3. Recreational activities in total weeks of life in cases and controls

	Total n=388	Cases n=194	Controls n=194	*p* value[#]
**Body covered**				
**Mountaineering** *(>2000m altitude;* total weeks, *mean* ± *SD*)				
All study participants	10 ± 37	10 ± 33	10 ± 40	0.969
Mountaineers only	42 ± 31	41 ± 27	42 ± 36	0.970
n (%)	92 (24)	46 (24)	46 (24)	
**Hiking** (total weeks, *mean* ± *SD*)				
All study participants	25 ± 80	25 ± 54	35 ± 105	0.239
Hikers only	50 ± 47	64 ± 32	134 ± 62	0.099
n (%)	231 (60)	122 (63)	109 (56)	
**Skiing/snowboarding** (total weeks, *mean* ± *SD)*				
All study participants	30 ± 43	30 ± 45	19 ± 41	**0.019**
Skiers/snowboarders only	41 ± 25	43 ± 22	38 ± 28	0.450
n (%)	233 (60)	134 (69)	99 (51)	**<0.001**
Non-skiers/snowboarders *(n (%))*	155 (40)	60 (31)	95 (49)	
**Body covered** (mountaineering, hiking, skiing; total weeks, *mean* ± *SD)*	64 ± 84	65 ± 90	64 ± 78	0.980
**Swim ware only** (total weeks, *mean* ± *SD)*				
**Watersports**				
All study participants	11 ± 25	11 ± 33	6 ± 17	0.052
Watersports-persons only	35 ± 15	39 ± 19	30 ± 11	0.300
n (%)	91 (24)	54 (28)	37 (19)	**0.042**
No water sport activities (*n (%)*)	297 (77)	140 (72)	157 (81)	
**Sunbathing**				
All study participants	31 ± 64	31 ± 59	36 ± 68	0.419
Sunbathers only	65 ± 28	55 ± 22	76 ± 34	0.051
n (%)	203 (52)	110 (57)	93 (48)	
**Swim ware only** (watersports, sunbathing; total weeks, *mean* ± SD)	42 ± 69	42 ± 72	42 ± 65	0.990
**uncovered body (nudist beach**; total weeks, *mean* ± *SD)*				
All study participants	4 ± 17	5 ± 23	3 ± 11	0.149
Nudists only	26 ± 12	30 ± 15	20 ± 8	0.310
n (%)	60 (16)	34 (18)	26 (13)	

[#] Significant *p* values (p<0.05) are displayed in bold. SD: standard deviation.

Table S4. Correlation between NMSC count and clinical/UV associated parameters

		NMSC	cSCC	BCC	BD	NMSC wo BD
**Clinical parameters**						
**Fitzpatrick skin type**	r_s	-0.094	-0.133	-0.030	-0.018	-0.107
FST I (1), FST II (2), FST III (3), FST IV (4)	***p* value**	0.191	0.064	0.674	0.803	0.136
**Hair color**	r_s	-0.029	0.013	-0.016	0.046	-0.042
Red (1), blond (2), brown (3), black (4)	***p* value**	0.687	0.855	0.822	0.520	0.557
**Eye color**	r_s	-0.187**	-0.087	-0.127	-0.074	-0.184*
Blue (1), green (2), grey (3), brown (4)	***p* value**	**0.009**	0.229	0.077	0.303	**0.010**
**Smoking**	r_s	0.022	0.067	-0.050	0.056	0.001
No (1), yes (2)	***p* value**	0.765	0.351	0.490	0.441	0.997
**Sunburn freckling**	r_s	0.057	0.154*	0.00	0.027	0.064
No (1), yes (2)	***p* value**	0.427	**0.033**	0.995	0.705	0.376
**Moles**	r_s	0.050	0.006	0.027	0.007	0.053
No (1), yes (2)	***p* value**	0.487	0.930	0.709	0.921	0.461
**Bald head** (Norwood scale, males only)	r_s	-0.014	0.075	-0.71	-0.034	-0.007
No (1), yes (2)	***p* value**	0.864	0.363	0.390	0.685	0.933
**Sunburns with skin peeling**						
Age 10-19	r_s	-0.017	0.083	-0.006	0.071	0.017
	***p* value**	0.813	0.251	0.936	0.322	0.813
Age 20-39	r_s	0.010	0.091	-0.028	-0.087	0.049
	***p* value**	0.885	0.208	0.698	0.230	0.495
Age 40-60	r_s	0.005	0.020	-0.003	-0.062	0.025
	***p* value**	0.945	0.785	0.966	0.392	0.725
Age >60	r_s	0.090	-0.022	0.130	0.004	0.115
	***p* value**	0.264	0.790	0.107	0.957	0.155
**Sunburns with skin blistering**						
Age 10-19	r_s	-0.028	0.013	-0.040	-0.003	-0.024
	***p* value**	0.700	0.861	0.582	0.967	0.742
Age 20-39	r_s	0.176*	0.185**	0.106	0.154*	0.178*
	***p* value**	**0.014**	**0.010**	0.141	**0.032**	**0.013**
Age 40-60	r_s	-0.010	-0.007	-0.050	0,061	-0.040
	***p* value**	0.894	0.919	0.486	0,671	0.577
Age >60	r_s / ***p* value**	-	-	-	-	-
**Main place of residence**						
**Altitude**	r_s	0.022	0.046	-0.019	0.087	0.015
	***p* value**	0.764	0.522	0.790	0.230	0.837
**Latitude**	r_s	0.025	-0.050	0.014	0.037	0.004
	***p* value**	0.726	0.488	0.842	0.613	0.959

Table S5. Reaction to sun exposure and sunburns in different periods of life

	Total n=388	Cases n=194	Controls n=194	*p* value
**Sunburns with skin peeling**				
**At age 10-19** *(n (%))*				
Total number of	388	194	194	
Yes 1-5	188 (48)	100 (52)	88 (45)	0.530
6-10	52 (13)	25 (13)	27 (14)	
11-20	9 (2)	5 (3)	4 (2)	
> 20	1 (0)	1 (1)	0	
None	138 (36)	63 (33)	75 (39)	
**At age 20-39** *(n (%))*				
Total number of	388	194	194	
Yes 1-5	151 (39)	82 (42)	69 (36)	0.236
6-10	27 (7)	10 (5)	17 (9)	
11-20	4 (1)	3 (2)	1 (1)	
> 20	0	0	0	
None	206 (53)	99 (51)	107 (55)	
**At age 40-59** *(n (%))*				
Total number of	386	193	193	
Yes 1-5	75 (19)	32 (17)	43 (22)	0.244
6-10	9 (2)	6 (3)	3 (2)	
11-20	0	0	0	
> 20	0	0	0	
None	302 (78)	155 (80)	147 (76)	
**At age ≥ 60** *(n (%))*				
Total number of	314	157	157	
Yes 1-5	8 (3)	4 (3)	4 (3)	0.978
6-10	0	0	0	
11-20	0	0	0	
> 20	0	0	0	
None	306 (98)	153 (98)	153 (98)	
**Sunburns with skin**				
**At age 10-19** *(n (%))*				
Total number of	388	194	194	
Yes 1-5	42 (11)	26 (13)	16 (8)	0.157
6-10	3 (1)	2 (1)	1 (1)	
11-20	2 (1)	2 (1)	0	
> 20	0	0	0	
None	341 (88)	164 (85)	177 (91)	
**At age 20-39** *(n (%))*				
Total number of	388	194	194	
Yes 1-5	31 (8.0)	17 (9)	14 (7)	0.089
6-10	5 (1)	5 (3)	0	
11-20	1 (1)	1 (1)	0	
> 20	0	0	0	
None	351 (91)	171 (88)	180 (93)	
**At age 40-59** *(n (%))*				
Total number of	386	193	193	
Yes 1-5	16 (4)	8 (4)	8 (4)	0.606
6-10	1 (0)	1 (1)	0	
11-20	0	0	0	
> 20	0	0	0	
None	369 (96)	184 (95)	185 (96)	
**At age ≥ 60** *(n (%))*				
Total number of	311	154	157	
Yes 1-5	1 (0)	0	1 (1)	0.321
6-10	0	0	0	
11-20	0	0	0	
> 20	0	0	0	
None	310 (100)	154 (100)	156 (99)	

$p<0.05$ is considered significant

Table S6. Characteristics of a subset of pairs additionally matched for Fitzpatrick skin type

	**Cases** (n=97)	**Controls** (n=97)	***p* value**
**Age at visit,** years *(mean ± SD)*	69 ± 8.9	69 ± 8.6	1
**Post-TX period,** years *(mean ± SD)*	12.2 ± 7.3	12.8 ± 8.9	0.995
**Total number NMSC,** *(mean ± SD)*	8 ± 13	0	**<0.001**
**Skin aging score,** *(mean ± SD)*			
ISA-score	6.31 ± 2.81	7.13 ± 2.70	**0.034**
ESA-score	11.71 ± 5.22	13.50 ± 5.21	**0.018**
TSA-score	18.02 ± 7.31	20.50 ± 7.11	**0.016**
	**Cases** n (%)	**Controls** n (%)	***p* value**
**Fitzpatrick skin type (FST)**			
FST II	43 (44)	43 (44)	1
FST III	54 (56)	54 (56)	1
**Type of OTR**			
HTR	48 (50)	48 (50)	1
KTR	32 (33)	32 (33)	1
LTR	8 (8)	8 (8)	1
LuTR	9 (9)	9 (9)	1
**Smoking history**			
Yes	57 (59)	72 (74)	0.060
No	40 (41)	25 (26)	

p<0.05 is considered significant, indicated in bold, SD=standard deviation, TX=transplantation, NMSC=non-melanoma skin cancer, HTR=heart transplant recipients, KTR=kidney transplant recipients, LTR=liver transplant recipients, LuTR=lung transplant recipients, ISA score=intrinsic skin aging score, ESA score=extrinsic skin aging score, TSA score=total skin aging score.

Table S7a. The number and percentage of the different tumor entities in different localizations

	Cases	Tumors	Localization				
			**Head/neck**	**Lips**	**Trunk**	**Upper ext.**	**Lower ext.**
	**Cases** n=194	**Tumors** n=1649	n=776	n=17	n=526	n=190	n=140
**NMSC** *(n (%))*							
**total**	**194 (100)**	**1633 (100)**	**767 (100)**	**17 (100)**	**521 (100)**	**189 (100)**	**139 (100)**
without BD	101 (87)	1353 (83)	661 (86)	17 (100)	435 (83)	144 (76)	96 (69)
**cSCC**	137 (71)	620 (38)	378 (49)	16 (94)	125 (24)	70 (37)	31 (22)
**BCC**	139 (72)	733 (45)	283 (37)	1 (6)	310 (60)	74 (39)	65 (47)
**BD**	93 (48)	280 (17)	106 (14)	0	86 (16)	45 (24)	43 (31)
**PDS**	3 (2)	3 (0.2)	3 (0.4)	0	0	0	0
**Melanoma**	12 (6)	13 (0.8)	6 (0.8)	0	5 (1)	1 (0.5)	1 (0.7)

The bold numbers indicate 100% of NMSC in the respective localization. NMSC=non-melanoma skin cancer,
cSCC=cutaneous squamous cell carcinoma, BCC=basal cell carcinoma, BD=Bowen´s disease, PDS=pleomorphic
dermal sarcoma, SD=standard deviation. The percentage of PDS and melanoma numbers refers to the total numbers
of tumors (n=1649).

Table S7b. Distribution of the tumors in different localizations

	**Cases** n=194	**Tumors** n=1649	**Localization**									
			**Head/neck**		**Lips**		**Trunk**		**Upper extremities**		**Lower extremities**	
			Tumors	Cases *(n (%))*	Tumors	Cases *(n (%))*	Tumors	Cases *(n (%))*	Tumors	Cases *(n (%))*	Tumors	Cases *(n (%))*
**NMSC** *(n (%))*												
**total**	**194**	**1633 (100)**	767 (47)	159 (82)	17 (1)	16 (8)	521 (32)	99 (51)	189 (12)	45 (23)	139 (9)	40 (21)
without BD	**101**	**1353 (100)**	661 (49)	79 (78)	17 (1)	7 (7)	435 (32)	45 (45)	144 (11)	15 (15)	96 (7)	8 (8)
**cSCC**	**137**	**620 (100)**	378 (61)	112 (82)	16 (3)	15 (11)	125 (20)	36 (26)	70 (11)	27 (20)	31 (5)	17 (12)
**BCC**	**139**	**733 (100)**	283 (39)	102 (73)	1 (0.4)	1 (1)	310 (42)	84 (60)	74 (10)	28 (20)	65 (9)	31 (22)
**BD**	**93**	**280 (100)**	106 (38)	56 (60)	0	0	86 (31)	39 (42)	45 (16)	31 (33)	43 (15)	30 (32)
**PDS**	**3**	**3 (100)**	3 (100)	3 (100)	0	0	0	0	0	0	0	0
**Melanoma**	**12**	**13 (100)**	6 (46)	6 (50)	0	0	5 (39)	4 (33)	1 (8)	1 (8)	1 (8)	1 (8)

NMSC=non-melanoma skin cancer, cSCC=cutaneous squamous cell carcinoma, BCC=basal cell carcinoma, BD=Bowen´s disease, PDS=pleomorphic dermal sarcoma.

Table S7c. Mean number of tumors per patient and tumor entity

Tumor type	Cases n=194 (%)	Number of tumors per patient *(mean ± SD)*
**NMSC**	194 (100)	8.42 ± 14.48
**cSCC**	34 (18)	1.88 ± 2.50
**BCC**	39 (20)	3.03 ± 4.81
**BD**	5 (3)	5.00 ± 5.83
**cSCC+BCC**	28 (14)	7.96 ± 16.69
**cSCC+BD**	16 (8)	5.38 ± 3.85
**BCC+BD**	13 (7)	6.08 ± 6.66
**cSCC+BCC+BD**	59 (30)	17.93 ± 22.16

NMSC=non-melanoma skin cancer, cSCC=cutaneous squamous cell carcinoma, BCC=basal cell carcinoma, BD=Bowen´s disease, SD=standard deviation

Table S8. Correlation between NMSC count and age, gender, aging score, time to first NMSC, number of AKs, and MC1R risk groups

		Number of NMSC
**Older age at visit**	r_s	0.314**
	***p* value**	**<0.001**
**Older age at TX (>60y)**	r_s	0.103
	***p* value**	0.152#
**Longer post-TX period**	r_s	0.185**
	***p* value**	**0.010**
**Gender**	r_s	-0.005
Female (1), male (2)	***p* value**	0.448
**Higher skin aging score**		
ISA-score	r_s	0.272**
	***p* value**	**<0.001**
ESA-score	r_s	0.280**
	***p* value**	**<0.001**
TSA-score	r_s	0.321**
	***p* value**	**<0.001**
**Time to first NMSC**	r_s	-0.168*
	***p* value**	**0.019**
**Actinic keratoses**	r_s	-0.154*
	***p* value**	**0.032**
***MC1R* risk groups**	r_s	0.204*
Low (1), medium+high (2)	***p* value**	**0.004**

Spearman´s rank correlation coefficient (r_s) was calculated to assess potential correlations between these variables. p<0.05 is considered significant, indicated in bold. #Older age at TX did not correlate with a higher NMSC count due to the fact that some patients transplanted at younger age (i.e. 44-50 years) developed 30-112 NMSC in their post-TX period. TX=transplantation, NMSC=non-melanoma skin cancer, MC1R=Melanocortin-1-Receptor, ISA score=intrinsic skin aging score, ESA score=extrinsic skin aging score, TSA score=total skin aging score.

Table S9. Skin aging scores in a subset of cases and controls additionally matched for Fitzpatrick skin type and the whole study population

	**Subset of cases and controls matched for FST** (n=194)						**Whole study population** (n=388)		
	**Total** (n=194)			**Total** (n=194)			**Total** (n=376)		
**Skin aging scores**	**Cases** (n=97)	**Controls** (n=97)	***p* value**	**FST II** (n=86)	**FST III** (n=108)	***p* value**	**FST II** (n=177)	**FST III** (n=199)	***p* value**
**ISA- core** *(mean±SD)*	6.25 ± 2.75	7.07 ± 2.65	**0.034**	6.64 ± 2.70	6.68 ± 2.75	0.927	6.20 ± 2.78	6.46 ± 2.74	0.361
**ESA score** *(mean±SD)*	11.68 ± 5.22	13.46 ± 5.24	**0.018**	13.47 ± 5.12	11.86 ± 5.34	**0.036**	12.76 ± 4.85	11.77 ± 5.05	0.053
**TSA score** *(mean±SD)*	18.02 ± 7.25	20.52 ± 7.07	**0.016**	20.08 ± 7.08	18.62 ± 7.35	0.164	18.95 ± 6.79	18.25 ± 7.08	0.331

p<0.05 is considered significant, indicated in bold. SD=standard deviation, FST=Fitzpatrick skin type, ISA score=intrinsic skin aging score, ESA score=extrinsic skin aging score, TSA score=total skin aging score.

Table S10. Skin aging score values in association with age and gender

	Skin aging signs	Localization	Age < 60 years n=78 *mean±SD*	Age ≥ 60 years n=310 *mean±SD*	*p* value	Males n=298 *mean±SD*	Females n=90 *mean±SD*	*p* value
**Intrinsic skin aging signs**	Lax appearance	Face and neck	1.05 ± 0.74	1.82 ± 0.86	**<0.001**	1.68 ± 0.88	1.62 ± 0.93	0.582
	Uneven pigmentation	Face	0.69 ± 1.27	1.34 ± 1.49	**0.001**	1.29 ± 1.49	0.93 ± 1.40	**0.045**
	Fine wrinkles	Around eye-led, cheeks	1.28 ± 0.80	1.76 ± 0.92	**<0.001**	1.60 ± 0.86	1.88 ± 1.05	**0.011**
	Benign skin tumors	Head and neck	0.59 ± 0.86	0.65 ± 0.74	0.525	0.72 ± 0.81	0.39 ± 0.55	**<0.001**
	Reduced fat tissue	Cheeks	0.56 ± 0.77	1.34 ± 0.88	**<0.001**	1.23 ± 0.88	1.03 ± 1.01	0.082
**ISA score**			4.18 ± 2.35	6.90 ± 2.59	**<0.001**	6.51 ± 2.70	5.86 ± 2.94	0.500
**Extrinsic skin aging signs**	Sunburn freckles	Shoulders	0.73 ± 1.00	1.04 ± 1.05	**0.020**	0.97 ± 1.04	1.01 ± 1.07	0.723
	Lentigines solaris	Average value forehead, cheeks, dorsum of forearms and hands	1.72 ± 0.90	1.49 ± 0.82	**0.030**	1.48 ± 0.85	1.70 ± 0.79	**0.032**
	Pigment change	Face vs less UV-exposed skin	0.56 ± 0.71	1.09 ± 0.94	**<0.001**	1.08 ± 0.93	0.68 ± 0.85	**<0.001**
	Change of skin phototype		0.14 ± 0.42	0.30 ± 0.62	**0.035**	0.29 ± 0.61	0.19 ± 0.47	0.170
	Yellowness	Face	0.10 ± 0.11	0.12 ± 0.39	**0.017**	0.11 ± 0.37	0.07 ± 0.29	0.339
	Pseudoscars	Face	0.14 ± 0.55	0.02 ± 0.17	**0.001**	0.06 ± 0.33	0.01 ± 0.11	0.192
	Coarse wrinkles	Average value forehead, crow´s feet, infraorbital, upper lip region, nasolabial	1.85 ± 0.72	2.06 ± 0.59	**0.007**	2.04 ± 0.58	1.93 ± 0.75	0.153
	Elastosis cutis	Face	0.64 ± 0.79	1.55 ± 0.97	**<0.001**	1.31 ± 0.94	1.54 ± 1.19	0.054
	Cutis rhomboidalis nuchae	Neck	0.15 ± 0.67	1.05 ± 1.43	**<0.001**	0.96 ± 1.40	0.57 ± 1.18	**0.015**
	M. Favre Racouchot	Face	0.04 ± 0.34	0.29 ± 0.89	**0.014**	0.26 ± 0.85	0.17 ± 0.69	0.332
	Dryness	Face, back of forearm	0.32 ± 0.57	0.35 ± 0.64	0.725	0.29 ± 0.58	0.53 ± 0.72	**0.001**
	Comedones	Periorbital	0.04 ± 0.20	0.29 ± 0.76	**0.005**	0.24 ± 0.57	0.22 ± 1.02	0.740
	Telangiectasis	Cheeks, nose	0.64 ± 0.80	1.13 ± 0.88	**<0.001**	1.07 ± 0.88	0.90 ± 0.91	0.108
	Permanent erythema	Neck, decollete	1.04 ± 1.14	1.51 ± 1.04	**<0.001**	1.51 ± 1.05	1.12 ± 0.99	**0.002**
	Actinic keratosis	Whole skin	0.85 ± 1.36	0.85 ± 1.35	0.975	0.84 ± 1.35	0.90 ± 1.38	0.693
**ESA score**			8.85 ± 4.00	13.11 ± 4.87	**<0.001**	12.49 ± 4.89	11.51 ± 5.33	0.105
**TSA score**			12.97 ± 5.13	20.05 ± 6.66	**<0.001**	19.02 ± 6.76	17.32 ± 7.57	**0.043**

$p<0.05$ is considered significant, indicated in bold; SD=standard deviation, ISA score=intrinsic skin aging score, ESA score=extrinsic skin aging score, TSA score=total skin aging score.

Table S11. Skin aging scores in association with smoking history in the whole study population, subsets of FST-matched cases/controls pairs and cases with the highest UV-burden

	**Whole study population** (n=388)									**Subset of cases and controls additionally matched for FST** (n=194									**Cases with the highest UV burden°** (n=97)		
**Skin aging scores**	**Smoking history**			**Smoking history** Yes (n=243)			No (n=145)			**Smoking history**			**Smoking history** Yes (n=129)			No (n=65)			**Smoking history**		
	**Yes** (n=243)	**No** (n=145)	***p* value**	**Cases** (n=111)	**Controls** (n=132)	***p* value**	**Cases** (n=62)	**Controls** (n=83)	***p* value**	**Yes** (n=129)	**No** (n=65)	***p* value**	**Cases** (n=57)	**Controls** (n=72)	***p* value**	**Cases** (n=40)	**Controls** (n=25)	***p* value**	**Yes** (n=62)	**No** (n=35)	***p* value**
**ISA score** *(mean±SD)*	6.68 ± 2.77	5.81 ± 2.68	**0.002**	6.33 ± 2.88	6.98 ± 2.65	0.071	5.31 ± 2.54	6.47 ± 2.73	**0.010**	7.01 ± 2.69	5.97 ± 2.66	**0.012**	6.47 ± 2.84	7.22 ± 2.57	0.311	5.55 ± 2.48	6.64 ± 2.86	0.109	7.12 ± 2.82	5.61 ± 1.91	**0.003**
**ESA score** *(mean±SD)*	12.84 ± 5.14	11.28 ± 4.62	**0.003**	12.51 ± 5.15	13.12 ± 5.14	0.360	11.22 ± 4.76	11.37 ± 4.47	0.843	13.24 ± 5.41	11.25 ± 4.81	**0.013**	12.26 ± 5.29	14.01 ± 5.42	0.068	10.85 ± 5.06	11.88 ± 4.41	0.405	14.19 ± 5.12	12.26 ± 4.90	0.072
**TSA score** *(mean±SD)*	19.56 ± 7.14	17.06 ± 6.42	**0.001**	18.93 ± 7.24	20.08 ± 7.04	0.209	16.53 ± 6.47	17.77 ± 6.33	0.250	20.30 ± 7.34	17.22 ± 6.66	**0.005**	19.16 ± 7.37	21.21 ± 7.24	0.115	16.40 ± 6.83	18.52 ± 6.29	0.214	21.40 ± 7.15	17.86 ± 6.11	**0.015**

p<0.05 is considered significant, indicated in bold; SD=standard deviation, FST=Fitzpatrick skin type, °fourth quartile of total UV burden, ISA score=intrinsic skin aging score, ESA score=extrinsic skin aging score, TSA score=total skin aging score.

Table S12. Mitochondrial DNA point heteroplasmy (PHP): number of mtDNA PHP in UV-exposed versus UV-unexposed skin and in cases versus controls

	**All samples** (n=23)	***p* value**	**Cases** (n=10)	**Controls** (n=13)	***p* value**
**UV exposed skin** *(mean ± SD)*	2.00 ± 1.68	**<0.001**	0	0.08 ± 0.28	1
**UV unexposed skin** *(mean ± SD)*	0.04 ± 0.21		2 ± 1.16	2 ± 2.04	0.393

Means were compared by univariate ANOVA. *p*<0.05 is considered significant, indicated in bold; SD=standard deviation.

Table S13. Quantification of UV-exposure in different scenarios and different subsets (cases versus controls)

UV-exposure	**Matched cases and controls according to skin aging score** (n=114)			**Subset of cases and controls additionally matched for FST** (n=194)						**Cases and matched controls without AKs** (n=102)		
	**Cases** (n=57)	**Controls** (n=57)	***p* value**	**Cases** (n=97)	**Controls** (n=97)	***p* value**	**FST II** (n=86)	**FST III** (n=108)	***p* value**	**Cases** (n=51)	**Controls** (n=51)	***p* value**
**Time spent outside with unintentional sun exposure** *(May-August)*												
**Outdoor occupation** Total hours *(mean ± SD)*	5329 ± 9519	5429 ± 8254	0.957	4581 ± 8382	6578 ± 10646	0.148	5413 ± 9348	5712 ± 9852	0.830	4224 ± 7751	6635 ± 11094	0.206
**Total time spent outside with unintentional sun exposure** (including occupation; **UV-scenario I+II**) Total hours *(mean ± SD)*	31214 ± 20978	30188 ± 18180	0.801	27362 ± 11338	30318 ± 15000	0.123	29044 ± 13294	28678 ± 13443	0.850	27121 ± 9376	30693 ± 15621	0.165
**Time spent outside with intentional sun exposure** *(9am-3pm, May-August)*												
**Central Europe (UV-scenario III)** Total hours *(mean ± SD)*	5416 ± 3861	5015 ± 3376	0.593	5935 ± 3715	5371 ± 4175	0.321	5484 ± 3836	5788 ± 4054	0.596	5624 ± 3378	4931 ± 3621	0.320
**Southern geographic regions (UV-scenario IV)** Total hours *(mean ± SD)*	2398 ± 2570	3000 ± 3215	0.319	3351 ± 2860	2220 ± 2192	**0.002**	2699 ± 2459	2854 ± 2724	0.682	3395 ± 3069	2170 ± 2215	**0.023**
**Total time spent outside with intentional sun exposure (UV-scenario III+IV)** Total hours *(mean ± SD)*	7814 ± 4339	8016 ± 4512	0.826	9287 ± 5033	7591 ± 4551	**0.015**	8184 ± 5016	8642 ± 4747	0.516	9019 ± 4812	7100 ± 4365	**0.037**

Mean lifetime hours ± SD were calculated with univariate ANOVA; $p<0.05$ is considered significant, indicated in bold. AKs=actinic keratosis, SD=standard deviation, FST=Fitzpatrick skin type.

Table S14. Correlations of the skin aging scores with UV-exposure scenarios in a subset of 97 cases/control pairs matched for Fitzpatrick skin type

		Skin aging scores		
		**ISA score**	**ESA score**	**TSA score**
**Time spent outside with unintentional sun exposure** *(May-August)*	r_s	-0.024	0.148*	0.095
**Outdoor occupation**	***p* value**	0.736	**0.040**	0.188
**Total time spent outside with unintentional sun exposure** (including occupation; **UV-scenario I+II**)	r_s	0.115	0.230**	0.196**
	***p* value**	0.110	**0.001**	**0.006**
**Time spent outside with intentional sun exposure** *(9am-3pm, May-August)*				
**Central Europe**	r_s	0.087	0.146*	0.141
**(UV-scenario III)**	***p* value**	0.229	**0.042**	0.050
**Southern geographic regions**	r_s	-0.026	-0.029	-0.044
**(UV-scenario IV)**	***p* value**	0.719	0.690	0.543
**Total time spent outside with intentional sun**	r_s	0.058	0.125	0.104
**exposure (UV-scenario III+IV)**	***p* value**	0.420	0.082	0.151
**Outdoor leisure time activities during vacation**				
**Body covered**	r_s	0.134	0.129	0.141*
**(mountaineering, hiking, skiing)**	***p* value**	0.062	0.074	**0.049**
**Wearing swimwear**	r_s	-0.022	-0.001	-0.007
**(water sports, sunbathing)**	***p* value**	0.761	0.987	0.923
**Full body uncovered (nudist beach)**	r_s	0.041	0.047	0.043
	***p* value**	0.568	0.516	0.551
**Sunbed use** *(yes/no)*	r_s	-0.159**	-0.080	-0.128*
	***p* value**	**0.001**	0.116	**0.014**
**Gardening**	r_s	0.161**	0.188**	0.205**
	***p* value**	**0.006**	**0.002**	**0.001**

$p<0.05$ is considered significant, indicated in bold; r_s=Spearman´s rank correlation coefficient, *correlation, **strong correlation. ISA score=intrinsic skin aging score, ESA score=extrinsic skin aging score, TSA score=total skin aging score

Table S15. Correlations between skin aging scores and NMSCs located in different regions

			cSCC						BCC						Bowen´s disease					
		**NMSC** all sites	**cSCC** all sites	**Head/ neck**	**Trunk**	**Upper extr.**	**Lower extr.**	**Lips**	**BCC** all sites	**Head/ neck**	**Trunk**	**Upper extr.**	**Lower extr.**	**Lips**	**BD** all sites	**Head/ neck**	**Trunk**	**Upper extr.**	**Lower extr.**	**Lips**
**ISA score**	r_s	0.272**	0.190**	0.162*	0.170*	0.048	0.129	0.003	0.202**	0.172*	0.178*	0.174*	0.131	-0.118	0.168*	0.090	0.118	0.092	0.079	-
	***p* value**	**<0.001**	**0.008**	**0.024**	**0.018**	0.503	0.072	0.970	**0.005**	**0.017**	**0.013**	**0.015**	0.068	0.101	**0.020**	0.211	0.100	0.201	0.276	
**ESA score**	r_s	0.280**	0.279**	0.224**	0.215**	0.194**	0.135	0.041	0.213**	0.196**	0.128	0.161*	0.086	0.006	0.136	0.060	0.093	0.143*	0.065	-
	***p* value**	**<0.001**	**<0.001**	**0.002**	**0.003**	**0.007**	0.061	0.569	**0.003**	**0.006**	0.074	**0.025**	0.235	0.929	0.059	0.406	0.197	**0.046**	0.365	
**TSA score**	r_s	0.321**	0.276**	0.233**	0.225**	0.151*	0.147*	0.030	0.241**	0.217**	0.172*	0.191**	0.121	-0.057	0.182*	0.094	0.133	0.151*	0.080	-
	***p* value**	**<0.001**	**<0.001**	**0.001**	**0.002**	**0.036**	**0.041**	0.675	**0.001**	**0.002**	**0.016**	**0.008**	0.093	0.433	**0.011**	0.191	0.065	**0.036**	0.266	

$p<0.05$ is considered significant, indicated in bold; r_s=Spearman´s rank correlation coefficient,*correlation, **strong correlation. NMSC=non-melanoma skin cancer, cSCC=cutaneous squamous cell carcinoma including Bowen´s carcinoma, BCC=basal cell carcinoma, extr.=extremity, ISA score=intrinsic skin aging score, ESA score=extrinsic skin aging score, TSA score=total skin aging score.

Table S16. Correlation of NMSC (localization) with UV exposure

			cSCC						BCC						Bowen´s disease					
		**NMSC** all sites	**cSCC** all sites	**Head/ neck**	**Trunk**	**Upper extr.**	**Lower extr.**	**Lips**	**BCC** all sites	**Head/ neck**	**Trunk**	**Upper extr.**	**Lower extr.**	**Lips**	**BD** all sites	**Head/ neck**	**Trunk**	**Upper extr.**	**Lower extr.**	**Lips**
**Time spent outside with unintentional sun exposure** *(May-August)*																				
Outdoor occupation	r_s	0.030	0.181*	0.228**	-0.054	-0.036	-0.136	0.021	-0.088	-0.001	-0.069	-0.113	-0.026	-0.053	-0.089	0.063	-0.088	-0.100	-0.169*	-.
	***p* value**	0.673	**0.012**	**0.001**	0.453	0.618	0.063	0.767	0.224	0.992	0.341	0.117	0.718	0.460	0.219	0.380	0.221	0.165	**0.018**	
**Total time spent outside with unintentional sun exposure** (including occupation; **UV- scenario I+II**)	r_s	0.135	0.245**	0.303**	-0.010	-0.042	0.023	-0.006	-0.033	0.074	-0.110	0.005	-0.005	0.034	-0.066	0.116	-0.077	-0.091	-0.138	-
	***p* value**	0.060	**0.001**	**<0.001**	0.887	0.562	0.751	0.930	0.643	0.302	0.126	0.948	0.946	0.637	0.363	0.107	0.285	0.206	0.055	-.
**Time spent outside with intentional sun exposure** *(9am-3pm, May-August)*																				
**Central Europe (UV-scenario III)**	r_s	0.218**	0.195**	0.167*	0.153*	-0.034	0.001	0.055	0.102	0.087	0.024	0.086	0.089	0.056	0.133	0.124	0.084	0.046	0.067	-
	***p* value**	**0.002**	**0.007**	**0.020**	**0.033**	0.640	0.987	0.445	0.156	0.229	0.736	0.233	0.219	0.439	0.065	0.084	0.246	0.520	0.353	
**Southern geographic regions (UV-scenario IV)**	r_s	0.078	-0.081	-0.189**	0.105	0.050	0.227**	-0.019	0.123	0.106	0.035	0.123	0.165*	-0.066	0.082	-0.019	0.024	0.055	0.125	-
	***p* value**	0.278	0.261	**0.008**	0.145	0.490	**0.001**	0.792	0.087	0.140	0.631	0.087	**0.021**	0.358	0.257	0.795	0.737	0.449	0.082	
**Total time spent outside with intentional sun exposure (UV-scenario III+IV)**	r_s	0.226**	0.142*	0.075	0.187**	-0.010	0.121	0.055	0.141*	0.140	0.013	0.134	0.161*	0.011	0.161*	0.105	0.071	0.084	0.104	-
	***p* value**	**0.002**	**0.049**	0.301	**0.009**	0.893	0.092	0.449	0.050	0.052	0.859	0.063	**0.025**	0.880	**0.025**	0.147	0.324	0.242	0.150	
**Outdoor leisure time activities during vacation**																				
**Body covered** (mountaineering, hiking, skiing)	r_s	0.012	0.015	0.002	0.028	-0.037	-0.018	0.102	0.044	0.018	0.011	0.107	-0.015	0.005	-0.105	-0.155*	-0.036	-0.107	-0.018	-.
	***p* value**	0.868	0.838	0.976	0.698	0.610	0.799	0.158	0.544	0.808	0.875	0.137	0.838	0.950	0.145	**0.031**	0.622	0.138	0.800	
**Wearing swimwear** (water sports, sunbathing)	r_s	-0.067	-0.017	-0.066	0.082	-0.032	-0.020	0.066	-0.045	0.010	-0.056	-0.068	0.013	-0.033	0.020	-0.044	-0.026	-0.036	0.011	-
	***p* value**	0.351	0.814	0.359	0.254	0.657	0.786	0.364	0.535	0.894	0.436	0.343	0.852	0.645	0.778	0.542	0.724	0.614	0.881	
**Full body uncovered** (nudist beach)	r_s	0.010	-0.003	-0.013	0.034	0.095	-0.031	-0.022	0.041	0.006	0.012	00.14	0.050	-0.033	-0.082	-0.059	-0.081	0.061	0.045	-
	***p* value**	0.895	0.966	0.861	0.642	0.190	0.668	0.762	0.567	0.937	0.869	0.051	0.486	0.648	0.255	0.412	0.260	0.396	0.536	
**Sunbed use** (*sun-bed users only*)	r_s	0.052	-0.087	0.015	-0.097	0.098	-0.025	-0.035	-0.035	0.203	-0.115	-0.002	0.023	-	-0.157	-0.030	0.021	-0.113	-0.319*	-
	***p* value**	0.749	0.593	0.929	0.553	0.548	0.880	0.828	0.831	0.210	0.482	0.991	0.887		0.334	0.853	0.898	0.486	**0.045**	
**Gardening**	r_s	0.036	0.048	0.088	-0.004	0.039	0.088	-0.099	0.019	-0.038	0.054	0.028	-0.004	-0.015	-0.035	0.002	-0.030	0.027	0.044	-
	***p* value**	0.619	0.504	0.220	0.954	0.593	0.222	0.170	0.793	0.602	0.454	0.701	0.952	0.836	0.627	0.973	0.682	0.713	0.547	

$p<0.05$ is considered significant, indicated in bold; r_s=Spearman´s rank correlation coefficient,*correlation, **strong correlation. NMSC=non-melanoma skin cancer, cSCC=cutaneous squamous cell carcinoma including Bowen´s carcinoma, BCC=basal cell carcinoma, extr.=extremity

Table S17. Comparison of the skin aging scores and UV- scenarios in cases ≥5 NMSC: FST II versus FST III subsets

	cases ≥5 NMSC		
	**FST II** (n=48)	**FST III** (n=35)	***p* value**
**Skin aging score**			
**ISA score** *(mean ± SD)*	6.62 ± 2.70	6.69 ± 2.75	0.933
**ESA score** *(mean ± SD)*	13.12 ± 5.16	11.86 ± 5.42	**0.042**
**TSA Score** *(mean ± SD)*	20.03 ± 7.12	18.72 ± 7.25	0.172
**Time spent outside with unintentional sun exposure** *(May-August)*			
**Outdoor occupation** Total hours *(mean ± SD)*	5399 ± 9348	5645 ± 9552	0.870
**Total time spent outside with unintentional sun exposure (UV-scenario I+II)** Total hours *(mean ± SD)*	29091 ± 13294	28766 ± 13344	0.833
**Time spent outside with intentional sun exposure** *(9am-3pm, May-August)*			
**Central Europe (UV-scenario III)** Total hours *(mean ± SD)*	5484 ± 3226	5718 ± 4054	0.612
**Southern geographic regions (UV-scenario IV)** Total hours *(mean ± SD)*	2699 ± 2321	2854 ± 2724	0.671
**Total time spent outside with intentional sun exposure (UV-scenario III+IV)** Total hours *(mean ± SD)*	8184 ± 5100	8612 ± 4117	0.536

Mean and standard deviation (SD) are shown. $p<0.05$ is considered significant, indicated in bold. NMSC=non-melanoma skin cancer, FST=Fitzpatrick skin type. ISA score=intrinsic skin aging score, ESA score=extrinsic skin aging score, TSA score=total skin aging score.

Table S18a. ***MC1R*** **variants and their frequency in cases versus controls**

MC1R **variants (n=31)**	**dbSNP number**	***MC1R*** **risk type**	**Haplotypes**	**Frequency cases** n=194 het (%)/hom (%)	**Frequency controls** n=194 het (%)/hom (%)	***p*** **value °**	**Frequency whole study population** n=388 (%) het (%)/hom (%)
no variants	-	0	1	41 (21)	49 (25)	0.712	90 (23)
c.-13C>T	rs375789795	0	1n	0/0	1 (0.5)/0	1	1 (0.3)/0
c.86dupA, p.Asn29Lysfs*14	rs796296176	R	11	0/0	1 (0.5)/0	1	1 (0.3)/0
c.136C>T, p.Leu46Phe	rs775806791	r	14	1 (0.5%)/0	0/0	1	1 (0.3)/0
c.178G>T, p.Val60Leu	rs1805005	r	3	34 (17.5)/7 (3.6)	37 (19.1)/3 (1.5)	0.4	71 (18)/10 (3)
c.200G>A, p.Arg67Gln	rs34090186	r	19	1 (0.5)/0	0/0	1	1 (0.3)/0
c.247T>C, p.Ser83Pro	rs34474212	R	16	0/0	1 (0.5)/0	1	1 (0.3)/0
c.252C>A, p.Asp84Glu	rs1805006	R	10	3 (1.5)/0	3 (1.5)/0	1	6 (1.5)/0
c.274G>A, p.Val92Met	rs2228479	r	2	31 (16)/1 (0.5)	39 (20.1)/2 (1.0)	0.4	70 (18)/3 (0.8)
c.284C>T, p.Thr95Met	rs34158934	0	1g	1 (0.5)/0	0/0	1	1 (0.3)/0
c.359T>C, p.Ile120Thr	rs33932559	r	18	0/0	1 (0.5)/0	1	1 (0.3)/0
c.366G>A, p.Val122=	rs372353477	0	1f	1 (0.5)/0	0/0	1	1 (0.3)/0
c.378C>T, p.Ser126=	rs1299958681	0	1k	1 (0.5)/0	0/0	1	1 (0.3)/0
c.425G>A, p.Arg142His	rs11547464	R	6	2 (1)/0	4 (2.1)/0	0.685	6 (1.5)/0
c.439T>C, p.Phe147Leu	rs1165997971	r	17	1 (0.5)/0	0/0	1	1 (0.3)/0
c.451C>T, p.Arg151Cys	rs1805007	R	5	22 (11.3)/0	15 (7.7)/0	0.3	37 (9.5)/0
c.464T>C, p.Ile155Thr	rs1110400	R	8	6 (3.1)/0	0/0	**0.03**	6 (1.5)/0
c.478C>T, p.Arg160Trp	rs1805008	R	4	27 (14)/1 (0.5)	22 (11.3)/0	0.5	49 (13)/1 (0.3)
c.488G>A, p.Arg163Gln	rs885479	r	9	14 (7)/1 (0.5)	12 (6.2)/1 (0.5)	0.836	26 (7)/2 (0.5)
c.496dupG, p.Ala166Glyfs*73	rs780875127	R	12	1 (0.5)/0	0/0	1	1 (0.3)/0
c.699G>A, p.Gln233=	rs146544450	0	1a	1 (0.5)/0	2 (1)/0	1	3 (0.8)/0
c.754delC, p.Leu252Serfs*62	rs761973172	R	13	0/0	1 (0.5)/0	1	1 (0.3)/0
c.880G>C, p.Asp294His	rs1805009	R	7	4 (2.1)/0	3 (1.5)/0	1	7 (1.8)/0

c.883C>A, p.Pro295Thr	--	R	15	0/0	1 (0.5)/0	1	1 (0.3)/0
c.897C>T, p.Ala299=	rs760953265	0	1l	1 (0.5)/0	0/0	1	1 (0.3)/0
c.942A>G, p.Thr314=	rs2228478	0	1d, 1i, 2, 8	38 (19.6)/1 (0.5)	44 (22.7)/2 (1)	0.627	82 (21)/3 (0.8)
c.948C>T , p.Ser316=	rs151318945	0	1m	1 (0.5)/0	0/0	1	1 (0.3)/0
c.*59C>G	rs938331667	0	1h	0/0	1 (0.5)/0	1	1 (0.3)/0
c.*69T>G	rs770382929	0	1j	1 (0.5)/0	0/0	1	1 (0.3)/0
c.*140A>G	rs3212369	0	1d, 1e, 2, 8, 10	56 (28.9)/4 (2.1)	66 (34)/5 (2.6)	0.496	122 (31)/9 (2)
c.*177G>C	rs150450096	0	1b	1 (0.5)/0	0/0	1	1 (0.3)/0
c.*212_*217delCAGTCG	--	0	1c	1 (0.5)/0	0/0	1	1 (0.3)/0

p<0.05 is considered significant, indicated in bold. °*P* value for cases versus controls. Variant nomenclature according to the HGVS (Human Genome Variation Society); reference sequences for *MC1R*: NM_002386.4, NG_012026.1.; hom=homozygous, het=heterozygous.

Table S18b. *MC1R* haplotypes and their frequency in cases and controls

Haplotype	*MC1R* risk type	Nucleotide	Amino acid	**Frequency cases** n=194 het (%)/hom (%)	**Frequency controls** n=194 het (%)/hom (%)	***p* value °**	**Frequency whole study population** n=388 (%) het (%)/hom (%)
1⁺	0			60 (30)/100 (52)	71 (37)/95 (49)	0.4	131 (34)/195(50)
2	r	c.274G>A<br>c.942A>G<br>c.*140A>G	p.Val92Met<br>p.Thr314=	31 (16)/1 (0.5)	39 (20.1)/2 (1.0)	0.4	70 (18)/3 (0.8)
3	r	c.178G>T	p.Val60Leu	34 (17.5)/10 (2.6)	37 (19.1)/3 (1.5)	0.4	71 (18)/13 (3)
4	R	c.478C>T	p.Arg160Trp	27 (14)/1 (0.5)	22 (11.3)/0	0.5	49 (13)/1 (0.3)
5	R	c.451C>T	p.Arg151Cys	22 (11.3)/0	15 (7.7)/0	0.3	37 (9.5)/0
6	R	c.425G>A	p.Arg142His	2 (1)/0	4 (2.1)/0	0.685	6 (1.5)/0
7	R	c.880G>C	p.Asp294His	4 (2.1)/0	3 (1.5)/0	1	7 (1.8)/0
8	R	c.464T>C<br>c.942A>G<br>c.*140A>G	p.Ile155Thr<br>p.Thr314=	6 (3.1)/0	0/0	**0.03**	6 (1.5)/0
9	r	c.488G>A	p.Arg163Gln	14 (7)/1 (0.5)	12 (6.2)/1 (0.5)	0.836	26 (7)/ 2 (0.5)
10	R	c.252C>A<br>c.*140A>G	p.Asp84Glu	3 (1.5)/0	3 (1.5)/0	1	6 (1.5)/0
11	R	c.86dupA	p.Asn29Lysfs*14	0/0	1 (0.5)/0	1	1 (0.3)/0
12	R	c.496dupG	p.Ala166Glyfs*73	1 (0.5)/0	0/0	1	1 (0.3)/0
13	R	c.754delC	p.Leu252Serfs*62	0/0	1 (0.5)/0	1	1 (0.3)/0
14	r	c.136C>T	p.Leu46Phe	1 (0.5%)/0	0/0	1	1 (0.3)/0
15	R	c.883C>A	p.Pro295Thr	0/0	1 (0.5)/0	1	1 (0.3)/0
16	R	c.247T>C	p.Ser83Pro	0/0	1 (0.5)/0	1	1 (0.3)/0
17	r	c.439T>C	p.Phe147Leu	1 (0.5)/0	0/0		1 (0.3)/0
18	r	c.359T>C	p.Ile120Thr	0/0	1 (0.5)/0	1	1 (0.3)/0
19	r	c.200G>A	p.Arg67Gln	1 (0.5)/0	0/0	1	1 (0.3)/0

1⁺=no *MC1R* variant or variants without effect on MC1R function, °*p* value for cases versus controls, $p<0.05$ is considered significant, indicated in bold.
hom=homozygous, het=heterozygous

Table S18c. ***MC1R*** **diplotypes and their frequency in cases and controls**

Diplotype	*MC1R* risk type	Cases n=194 (%)	Controls n=194 (%)	Total n=388 (%)
1/1	0/0	60 (31)	71 (37)	131 (34)
1/2	0/r	22 (12)	27 (14)	49 (13)
1/3	0/r	27 (14)	32 (16)	59 (15)
1/4	0/R	18 (9)	11 (6)	29 (8)
1/5	0/R	16 (9)	9 (5)	25 (6)
1/6	0/R	1 (1)	2 (1)	3 (1)
1/7	0/R	2 (1)	0	2 (1)
1/8	0/R	2 (1)	0	2 (1)
1/9	0/r	8 (4)	9 (5)	17 (4)
1/10	0/R	2 (1)	2 (1)	4 (1)
1/11	0/R	0	1 (1)	1 (0.3)
1/12	0/R	1 (1)	0	1 (0.3)
1/13	0/R	0	1 (1)	1 (0.3)
1/16	0/R	0	1 (1)	1 (0.3)
1/17	0/r	1 (1)	0	1 (0.3)
2/2	rr	1 (1)	2 (1)	3 (1)
2/3	rr	3 (2)	2 (1)	5 (1)
2/4	Rr	2 (1)	5 (2)	7 (2)
2/5	Rr	2 (1)	3 (2)	5 (1)
2/7	Rr	0	2 (1)	2 (1)
2/8	Rr	1 (1)	0	1 (0.3)
2/9	rr	1 (1)	0	1 (0.3)
3/3	rr	7 (4)	2 (1)	9 (2)
3/4	Rr	1 (1)	1 (1)	2 (1)
3/5	Rr	1 (1)	0	1 (0.3)
3/7	Rr	0	1 (1)	1 (0.3)
3/9	rr	1 (1)	1 (1)	2 (1)
3/14	rr	1 (1)	0	1 (0.3)
3/18	rr	0	1 (1)	1 (0.3)
4/4	RR	1 (1)	0	1 (0.3)
4/5	RR	1 (1)	2 (1)	3 (1)
4/6	RR	1 (1)	2 (1)	3 (1)
4/8	RR	1 (1)	0	1 (0.3)
4/9	Rr	2 (1)	1 (1)	3 (1)
4/10	RR	1 (1)	0	1 (0.3)
4/19	Rr	1 (1)	0	1 (0.3)
5/7	RR	1 (1)	0	1 (0.3)
5/9	Rr	1 (1)	0	1 (0.3)
5/15	RR	0	1 (1)	1 (0.3)
7/8	RR	1 (1)	0	1 (0.3)
8/9	Rr	1 (1)	0	1 (0.3)
9/9	rr	1 (1)	1 (1)	2 (1)
9/10	Rr	0	1 (1)	1 (0.3)

Table S19. Association of *MC1R* risk groups with skin type, hair and eye color in cases versus controls

	MC1R						*MC1R*			*MC1R*	
	0/0	0/r	0/R	R/r	r/r	R/R	low	medium	high	Low	medium +high
**Fitzpatrick skin type**											
**Pale white skin (I)**											
Total n=9	0	1 (11)	0	0	0	8 (89)	1 (11)	0	8 (89)	1 (11)	8 (89)
Cases n=4	0	1 (25)	0	0	0	3 (75)	1 (25)	0	3 (75)	1 (25)	3 (75)
Controls n=5	0	0	0	0	0	5 (100)	0	0	5 (100)	0	5 (100)
***p* value**	0.236						0.236			0.236	
**Fair skin (II)**											
Total n=177	53 (30)	57 (32)	39 (22)	12 (7)	12 (7)	4 (2)	110 (62)	63 (36)	4 (2)	110 (62)	67 (38)
Cases n=107	27 (25)	33 (31)	29 (27)	5 (5)	9 (8)	4 (4)	60 (56)	43 (40)	4 (4)	60 (56)	47 (44)
Controls n=70	26 (37)	24 (34)	10 (15)	7 (10)	3 (4)	0	50 (71)	20 (29)	0	50 (71)	20 (29)
***p* value**	0.056						0.054			**0.028**	
**Darker white skin (III)**											
Total n=199	76 (38)	68 (34)	29 (15)	14 (7)	12 (6)	0	144 (72)	55 (28)	0	144 (72)	55 (28)
Cases n=83	33 (40)	24 (29)	13 (16)	7 (8)	6 (7)	0	57 (69)	26 (31)	0	57 (69)	26 (31)
Controls n=116	43 (37)	44 (38)	16 (14)	7 (6)	6 (5)	0	87 (75)	29 (25)	0	87 (75)	29 (25)
***p* value**	0.719						0.325			0.325	
**Light brown skin (IV)**											
Total n=3	2 (67)	0	1 (33)	0	0	0	2 (67)	1 (33)	0	2 (67)	1 (33)
Cases n=0	0	0	0	0	0	0	0	0	0	0	0
Controls n=3	2 (67)	0	1 (33)	0	0	0	2 (67)	1 (33)	0	2 (67)	1 (33)
***p* value**	-						-			-	
**Hair color**											
**Red**											
Total n=7	0	0	0	0	0	7 (100)	0	0	7 (100)	0	7 (100)
Cases n=2	0	0	0	0	0	2 (100)	0	0	2 (100)	0	2 (100)
Controls n=5	0	0	0	0	0	5 (100)	0	0	5 (100)	0	5 (100)
***p* value**	-						-			-	
**Blond**											
Total n=117	38 (33)	36 (31)	24 (20)	7 (6)	8 (7)	4 (3)	74 (63)	39 (33)	4 (3)	74 (64)	43 (36)
Cases n=74	22 (30)	21 (28)	18 (24)	2 (3)	7 (10)	4 (5)	43 (58)	27 (37)	4 (5)	43 (58)	31 (42)
Controls n=43	16 (37)	15 (35)	6 (14)	5 (12)	1 (2)	0	31 (72)	12 (28)	0	31 (72)	12 (28)
***p* value**	0.069						0.152			0.130	
**Light brown**											
Total n=100	28 (28)	31 (31)	23 (23)	9 (9)	8 (8)	1 (1)	59 (59)	40 (40)	1 (1)	59 (59)	41 (41)
Cases n=60	14 (23)	18 (30)	16 (27)	6 (10)	5 (8)	1 (2)	32 (53)	27 (45)	1 (2)	32 (53)	28 (47)
Controls n=40	14 (35)	13 (33)	7 (18)	3 (7)	3 (7)	0	27 (68)	13 (33)	0	27 (68)	13 (33)
***p* value**	0.708						0.298			0.158	
**Dark brown**											

Total n=136	57 (42)	44 (32)	19 (14)	9 (7)	7 (5)	0	101 (74)	35 (26)	0	101 (74)	35 (26)
Cases n=52	21 (40)	17 (33)	7 (13)	4 (8)	3 (6)	0	38 (73)	14 (27)	0	38 (73)	14 (27)
Controls n=84	36 (43)	27 (32)	12 (14)	5 (6)	4 (5)	0	63 (75)	21 (25)	0	63 (75)	21 (25)
***p* value**	0.991						0.803			0.803	
**Black**											
Total n=28	8 (28)	15 (53)	3 (11)	1 (4)	1 (4)	0	23 (82)	5 (18)	0	23 (82)	5 (18)
Cases n=6	3 (50)	2 (33)	1 (17)	0	0	0	5 (83)	1 (17)	0	5 (83)	1 (17)
Controls n=22	5 (23)	12 (54)	2 (9)	1 (5)	2 (9)	0	17 (77)	5 (23)	0	17 (77)	5 (23)
***p* value**	0.625						0.932			0.932	
**Eye color**											
**Blue**											
Total n=148	49 (33)	45 (31)	32 (22)	5 (3)	11 (7)	6 (4)	94 (64)	48 (32)	6 (4)	94 (64)	54 (36)
Cases n=92	23 (25)	29 (32)	25 (27)	3 (3)	9 (10)	3 (3)	52 (57)	37 (40)	3 (3)	52 (57)	40 (43)
Controls n=56	26 (46)	16 (29)	7 (12)	2 (4)	2 (4)	3 (5)	42 (75)	11 (20)	3 (5)	42 (75)	14 (25)
***p* value**	0.060						**0.034**			**0.024**	
**Grey**											
Total n=56	17 (30)	24 (43)	8 (14)	5 (9)	2 (4)	0	41 (73)	15 (27)	0	41 (73)	15 (27)
Cases n=24	7 (29)	10 (42)	5 (21)	1 (4)	1 (4)	0	17 (71)	7 (29)	0	17 (71)	7 (29)
Controls n=32	10 (31)	14 (44)	3 (9)	4 (13)	1 (3)	0	24 (75)	8 (25)	0	24 (75)	8 (25)
***p* value**	0.662						0.728			0.728	
**Green**											
Total n= 51	21 (41)	12 (23)	8 (16)	5 (10)	3 (6)	2 (4)	33 (65)	16 (31)	2 (4)	33 (65)	18 (35)
Cases n=32	16 (50)	6 (19)	5 (16)	2 (6)	2 (6)	1 (3)	22 (69)	9 (28)	1 (3)	22 (69)	10 (31)
Controls n=19	5 (26)	6 (32)	3 (16)	3 (16)	1 (5)	1 (5)	11 (58)	7 (37)	1 (5)	11 (58)	8 (42)
***p* value**	0.590						0.724			0.433	
**Brown**											
Total n=133	44 (33)	45 (34)	21 (16)	11 (8)	8 (6)	4 (3)	89 (67)	40 (30)	4 (3)	89 (67)	44 (33)
Cases n=46	14 (30)	13 (28)	7 (15)	6 (13)	3 (7)	3 (7)	27 (59)	16 (35)	3 (6)	27 (59)	19 (41)
Controls n=87	30 (34)	32 (37)	14 (16)	5 (6)	5 (6)	1 (1)	62 (71)	24 (28)	1 (1)	62 (71)	25 (29)
***p* value**	0.340						0.128			0.186	

p values were calculated using the chi square test, $p<0.05$ is considered significant, indicated in bold.

Table S20. ***MC1R*** **risk groups in different subsets of cases and controls**

	Cases versus controls				Cases: 1-4 vs ≥5 NMSC				Cases: 1-9 vs ≥10 NMSC			Cases ≥5 NMSC vs matched controls				Cases ≥10 NMSC vs matched controls			
***MC1R* risk groups**	**Total** n=388 (%)	**Cases** n=194 (%)	**Controls** n=194 (%)	***p* value**	**Total** n=194 (%)	**Cases 1-4** n= 107 (%)	**Cases ≥5** n=87 (%)	***p* value**	**Cases 1-9** n=150 (%)	**Cases ≥10** n=44 (%)	***p* value**	**Total** n=174 (%)	**Cases ≥5** n=87 (%)	**Matched controls** n=87 (%)	***p* value**	**Total** n=88 (%)	**Cases ≥10** n=44 (%)	**Matched controls** n=44 (%)	***p* value**
**0/0**	131 (34)	60 (31)	71 (36)		60 (31)	38 (35)	22 (25)		54 (36)	6 (14)		55 (31)	22 (25)	33 (39)		20 (23)	6 (14)	14 (32)	
**0/r**	126 (32)	58 (30)	68 (35)	0.970	58 (30)	36 (34)	22 (25)	0.887	46 (31)	12 (27)	0.113	50 (29)	22 (25)	28 (32)	0.737	27 (31)	12 (27)	15 (34)	0.221
**0/R**	69 (18)	42 (22)	27 (14)	**0.044**	42 (22)	19 (18)	23 (26)	0.072	29 (19)	13 (30)	**0.010**	38 (22)	23 (27)	15 (17)	0.081	24 (27)	13 (30)	11 (25)	0.087
**R/r**	26 (7)	12 (6)	14 (7)	0.974	12 (6)	7 (6)	5 (6)	0.744	7 (4)	5 (12)	**0.010**	9 (5)	5 (6)	4 (5)	0.410	7 (8)	5 (12)	2 (5)	0.058
**r/r**	24 (6)	15 (8)	9 (5)	0.137	15 (8)	6 (6)	9 (10)	0.107	10 (7)	5 (12)	**0.031**	13 (8)	9 (11)	4 (5)	0.073	7 (8)	5 (12)	2 (5)	0.058
**R/R**	12 (3)	7 (4)	5 (3)	0.409	7 (4)	1 (1)	6 (7)	**0.036**	4 (3)	3 (7)	**0.029**	9 (5)	6 (7)	3 (3)	0.160	3 (3)	3 (7)	0	0.999
**low risk** (0/0, 0/r)	257 (66)	118 (61)	139 (71)	0.096	118 (61)	74 (69)	44 (51)	**0.004**	100 (67)	18 (41)	**0.004**	105 (60)	44 (51)	61 (70)	**0.030**	47 (53)	18 (41)	9 (66)	**0.027**
**medium risk** (r/r, 0/R, R/r)	119 (31)	69 (36)	50 (26)		69 (36)	32 (30)	37 (42)		45 (30)	23 (52)		60 (35)	37 (43)	23 (27)		38 (43)	23 (52)	15 (34)	
**high risk** (R/R)	12 (3)	7 (3.5)	5 (3)		7 (4)	1 (1)	6 (7)		4 (3)	3 (7)		9 (5)	6 (7)	3 (4)		3 (3)	3 (7)	0	
**low risk** (0/0, 0/r)	257 (66)	118 (61)	139 (71)	**0.030**	118 (61)	74 (69)	44 (51)	**0.004**	100 (67)	18 (41)	**0.001**	105 (60)	44 (51)	61 (70)	**0.008**	47 (53)	18 (41)	29 (66)	**0.019**

**medium + high risk** (r/r, 0/R, R/r, RR)	128 (34)	76 (39)	55 (29)		76 (39)	33 (31)	43 (49)		50 (33)	26 (59)		69 (40)	43 (50)	26 (30)		41 (47)	26 (59)	15 (34)	

$p<0.05$ is considered significant, indicated in bold, NMSC=non-melanoma skin cancer.

Table S21. Association of *MC1R* risk groups with skin pigmentation in different subgroups of OTR

	MC1R						*MC1R*			*MC1R*	
	**0/0**	**0/r**	**0/R**	**R/r**	**r/r**	**R/R**	**Low**	**Medium**	**High**	**Low**	**Medium + high**
**Cases ≥5 NMSC and matched controls**											
**Pale white skin (FST I)**											
Total, n=7	0	1 (14)	0	0	0	6 (86)	1 (14)	0	6 (86)	1 (14)	6 (86)
Cases ≥5, n=4	0	1 (25)	0	0	0	3 (75)	1 (25)	0	3 (75)	1 (25)	3 (75)
Controls, n=3	0	0	0	0	0	3 (100)	0	0	3 (100)	0	3 (100)
***p* value**	0.350						0.350			0.350	
**Fair skin (FST II)**											
Total, n=82	24 (29)	24 (29)	21 (26)	4 (5)	6 (7)	3 (4)	48 (58)	31 (38)	3 (4)	48 (58)	34 (41)
Cases ≥5, n=49	11 (22)	12 (25)	16 (33)	2 (4)	5 (10)	3 (6)	23 (47)	23 (47)	3 (6)	23 (47)	26 (53)
Controls, n=33	13 (39)	12 (37)	5 (15)	2 (6)	1 (3)	0	25 (76)	8 (24)	0	25 (76)	8 (24)
***p* value**	0.117						**0.023**			**0.008**	
**Darker white skin (FST III)**											
Total n=85	31 (37)	25 (29)	17 (20)	5 (6)	7 (8)	0	56 (66)	29 (34)	0	56 (66)	29 (34)
Cases ≥5, n=34	11 (32)	9 (27)	7 (21)	3 (9)	4 (12)	0	20 (59)	14 (41)	0	20 (59)	14 (41)
Controls, n=51	20 (39)	16 (31)	10 (20)	2 (4)	3 (6)	0	36 (71)	15 (29)	0	36 (71)	15 (29)
***p* value**	0.712						0.262			0.187	
**Light brown skin (FST IV)**											
Total, n=0	0	0	0	0	0	0	0	0	0	0	0
Cases ≥5, n=0	0	0	0	0	0	0	0	0	0	0	0
Controls, n=0	0	0	0	0	0	0	0	0	0	0	0
***p* value**	-						-			-	
**Cases ≥10 NMSC and matched controls**											
**Pale white skin (FST I)**											
Total, n=2	0	1 (50)	0	0	0	1 (50)	1 (50)	0	1 (50)	1 (50)	1 (50)
Cases ≥10, n=2	0	1 (50)	0	0	0	1 (50)	1 (50)	0	1 (50)	1 (50)	1 (50)
Controls, n=0	0	0	0	0	0	0	0	0	0	0	0
***p* value**	-						-			-	
**Fair skin (FST II)**											
Total, n=45	10 (22)	13 (29)	14 (31)	3 (7)	3 (7)	2 (4)	23 (51)	20 (45)	2 (4)	23 (51)	22 (49)
Cases ≥10, n=27	4 (16)	7 (26)	10 (37)	2 (7)	2 (7)	2 (7)	11 (41)	14 (52)	2 (7)	11 (41)	16 (59)
Controls, n=18	6 (33)	6 (33)	4 (22)	1 (6)	1 (6)	0	12 (67)	6 (33)	0	12 (67)	6 (33)
***p* value**	0.538						0.166			0.088	
**Darker white skin (FST III)**											
Total, n=41	11 (27)	12 (29)	10 (24)	4 (10)	4 (10)	0	23 (56)	18 (44)	0	23 (56)	18 (44)
Cases ≥10, n=15	2 (13)	4 (27)	3 (20)	3 (20)	3 (20)	0	6 (40)	9 (60)	0	6 (40)	9 (60)
Controls, n=26	9 (34)	8 (31)	7 (27)	1 (4)	1 (4)	0	17 (66)	9 (35)	0	17 (65)	9 (35)
***p* value**	0.139						0.115			0.115	
**Light brown skin (FST IV)**											
Total, n=0	0	0	0	0	0	0	0	0	0	0	0
Cases ≥10, n=0	0	0	0	0	0	0	0	0	0	0	0
Controls, n=0	0	0	0	0	0	0	0	0	0	0	0
***p* value**	-						-			-	
**Cases ≥5 NMSC and cases 1-4 NMSC**											

**Pale white skin (FST I)**											
Total, n=4	0	1 (25)	0	0	0	3 (75)	1 (25)	0	3 (75)	1 (25)	3 (75)
Cases ≥5, n=4	0	1 (25)	0	0	0	3 (75)	1 (25)	0	3 (75)	1 (25)	3 (75)
Cases 1-4, n=0	0	0	0	0	0	0	0	0	0	0	0
***p* value**	-						-			-	
**Fair skin (FST II)**											
Total, n=107	27 (25)	33 (31)	29 (27)	5 (5)	9 (8)	4 (4)	60 (56)	43 (40)	4 (4)	60 (56)	47 (44)
Cases ≥5, n=49	11 (23)	12 (25)	16 (33)	2 (4)	5 (10)	3 (6)	23 (47)	23 (47)	3 (6)	23 (47)	26 (53)
Cases 1-4, n=58	16 (28)	21 (36)	13 (22)	3 (5)	4 (7)	1 (2)	37 (64)	20 (34)	1 (2)	37 (64)	21 (36)
***p* value**	0.511						0.154			0.060	
**Darker white skin (FST III)**											
Total, n=83	33 (40)	24 (29)	13 (16)	7 (8)	6 (7)	0	57 (69)	26 (31)	0	57 (69)	26 (31)
Cases ≥5, n=34	11 (32)	9 (26)	7 (21)	3 (9)	4 (12)	0	20 (59)	14 (41)	0	20 (59)	14 (41)
Cases 1-4, n=49	22 (45)	15 (31)	6 (12)	4 (8)	2 (4)	0	37 (76)	12 (24)	0	37 (76)	12 (24)
***p* value**	0.485						0.107			0.107	
**Light brown skin (FST IV)**											
Total, n=0	0	0	0	0	0	0	0	0	0	0	0
Cases ≥5, n=0	0	0	0	0	0	0	0	0	0	0	0
Cases 1-4, n=0	0	0	0	0	0	0	0	0	0	0	0
***p* value**	-						-			-	
**Cases ≥10 NMSC and cases 1-9 NMSC**											
**Pale white skin (FST I)**											
Total, n=4	0	1 (25)	0	0	0	3 (75)	1 (25)	0	3 (75)	1 (25)	3 (75)
Cases ≥10, n=2	0	1 (50)	0	0	0	1 (50)	1 (50)	0	1 (50)	1 (50)	1 (50)
Cases 1-9, n=2	0	0	0	0	0	2 (100)	0	0	2 (100)	0	2 (100)
***p* value**	0.248						0.248			0.248	
**Fair skin (FST II)**											
Total, n=107	27 (25)	33 (31)	29 (27)	5 (5)	9 (8)	4 (4)	60 (56)	43 (40)	4 (4)	60 (56)	47 (44)
Cases ≥10, n=27	4 (16)	7 (26)	10 (37)	2 (7)	2 (7)	2 (7)	11 (41)	14 (52)	2 (7)	11 (41)	16 (59)
Cases 1-9, n=80	23 (29)	26 (32)	19 (24)	3 (4)	7 (9)	2 (2)	49 (61)	29 (36)	2 (3)	49 (61)	31 (39)
***p* value**	0.407						0.133			0.075	
**Darker white skin (FST III)**											
Total, n=83	33 (40)	24 (29)	13 (16)	7 (8)	6 (7)	0	57 (69)	26 (31)	0	57 (69)	26 (31)
Cases ≥10, n=15	2 (13)	4 (27)	3 (20)	3 (20)	3 (20)	0	6 (40)	9 (60)	0	6 (40)	9 (60)
Cases 1-9, n=68	31 (46)	20 (29)	10 (15)	4 (6)	3 (4)	0	51 (75)	17 (25)	0	51 (75)	17 (25)
***p* value**	**0.033**						**0.008**			**0.008**	
**Light brown skin (FST IV)**											
Total, n=0	0	0	0	0	0	0	0	0	0	0	0
Cases ≥10, n=0	0	0	0	0	0	0	0	0	0	0	0
Cases 1-9, n=0	0	0	0	0	0	0	0	0	0	0	0
***p* value**	-						-			-	

p values were calculated using the chi-square test, $p<0.05$ is considered significant, significant values are indicated in bold script, FST=Fitzpatrick skin type, NMSC=non-melanoma skin cancer.

Table S22. Association of *MC1R* risk groups with hair color in different subgroups of OTR

Hair color	*MC1R* 0/0	0/r	0/R	R/r	r/r	R/R	*MC1R* Low	Medium	High	*MC1R* Low	Medium + high
**Cases ≥5 NMSC and matched controls**											
**Red**											
Total, n=5	0	0	0	0	0	5 (100)	0	0	5 (100)	0	5 (100)
Cases ≥5, n=2	0	0	0	0	0	2 (100)	0	0	2 (100)	0	2 (100)
Controls, n=3	0	0	0	0	0	3 (100)	0	0	3 (100)	0	3 (100)
***p* value**	-						-			-	
**Blond**											
Total, n=51	16 (31)	12 (24)	12 (24)	2 (4)	5 (10)	4 (8)	28 (55)	19 (37)	4 (8)	28 (55)	23 (45)
Cases ≥5, n=34	10 (29)	5 (15)	9 (26)	1 (3)	5 (15)	4 (12)	15 (44)	15 (44)	4 (12)	15 (44)	19 (56)
Controls, n=17	6 (35)	7 (41)	3 (18)	1 (6)	0	0	13 (77)	4 (24)	0	13 (77)	4 (24)
***p* value**	0.125						0.066			0.058	
**Light brown**											
Total, n=46	16 (35)	12 (26)	11 (24)	3 (6)	4 (9)	0	28 (61)	18 (39)	0	28 (61)	18 (39)
Cases ≥5, n=24	6 (25)	6 (25)	7 (29)	2 (8)	3 (13)	0	12 (50)	12 (50)	0	12 (50)	12 (50)
Controls, n=22	10 (46)	6 (27)	4 (18)	1 (5)	1 (5)	0	16 (73)	6 (27)	0	16 (73)	6 (27)
***p* value**	0.546						0.115			0.115	
**Dark brown**											
Total, n=62	22 (35)	21 (34)	13 (21)	3 (5)	3 (5)	0	43 (69)	19 (31)	0	43 (69)	19 (31)
Cases ≥5, n=25	6 (24)	10 (40)	6 (24)	2 (8)	1 (4)	0	16 (64)	9 (36)	0	16 (64)	9 (36)
Controls, n=37	16 (43)	11 (30)	7 (19)	1 (3)	2 (5)	0	27 (73)	10 (27)	0	27 (73)	10 (27)
***p* value**	0.536						0.452			0.337	
**Black**											
Total, n=10	1 (10)	5 (50)	2 (20)	1 (10)	1 (10)	0	6 (60)	4 (40)	0	6 (60)	4 (40)
Cases ≥5, n=2	0	1 (50)	1 (50)	0	0	0	1 (50)	1 (50)	0	1 (50)	1 (50)
Controls, n=8	1 (13)	4 (50)	1 (13)	1 (13)	1 (13)	0	5 (63)	3 (38)	0	5 (63)	3 (38)
***p* value**	0.759						0.747			0.747	
**Cases ≥10 NMSC and matched controls**											
**Red**											
Total, n=1	0	0	0	0	0	1 (100)	0	0	1 (100)	0	1 (100)
Cases ≥10, n=1	0	0	0	0	0	1 (100)	0	0	1 (100)	0	1 (100)
Controls, n=0	0	0	0	0	0	0	0	0	0	0	0
***p* value**	-						-			-	
**Blond**											
Total, n=28	7 (25)	9 (32)	6 (22)	2 (7)	2 (7)	2 (7)	16 (57)	10 (36)	2 (7)	16 (57)	12 (43)
Cases ≥10, n=17	4 (24)	4 (23)	4 (23)	1 (7)	2 (12)	2 (12)	8 (47)	7 (41)	2 (12)	8 (47)	9 (53)
Controls, n=11	3 (27)	5 (46)	2 (18)	1 (9)	0	0	8 (73)	3 (27)	0	8 (73)	3 (27)
***p* value**	0.577						0.297			0.180	
**Light brown**											
Total, n=22	7 (32)	4 (18)	6 (27)	2 (9)	3 (14)	0	11 (50)	11 (50)	0	11 (50)	11 (50)
Cases ≥10, n=12	1 (8)	3 (25)	4 (33)	2 (17)	2 (17)	0	4 (33)	8 (67)	0	4 (33)	8 (67)
Controls, n=10	6 (60)	1 (10)	2 (20)	0	1 (10)	0	7 (70)	3 (30)	0	7 (70)	3 (30)
***p* value**	0.114						0.087			0.087	
**Dark brown**											
Total, n=31	7 (23)	10 (32)	11 (36)	2 (6)	1 (3)	0	17 (55)	14 (45)	0	17 (55)	14 (45)
Cases ≥10, n=13	1 (8)	4 (31)	5 (38)	2 (15)	1 (8)	0	5 (39)	8 (61)	0	5 (39)	8 (61)
Controls, n=18	6 (33)	6 (33)	6 (33)	0	0	0	12 (67)	6 (33)	0	12 (67)	6 (33)
***p* value**	0.170						0.119			0.119	
**Black**											
Total, n=6	0	3 (50)	1 (17)	1 (17)	1 (17)	0	3 (50)	3 (50)	0	3 (50)	3 (50)
Cases ≥10, n=1	0	1 (100)	0	0	0	0	1 (100)	0	0	1 (100)	0

Controls, n=5	0	2 (40)	1 (20)	1 (20)	1 (20)	0	2 (40)	3 (60)	0	2 (40)	3 (60)
***p* value**	0.753						0.273			0.273	

p values were calculated using the chi-square test, $p<0.05$ is considered significant, indicated in bold, NMSC=non-melanoma skin cancer.

Table S23. Association of *MC1R* risk groups with eye color in different subgroups of OTR

	MC1R						*MC1R*			*MC1R*	
	**0/0**	**0/r**	**0/R**	**R/r**	**r/r**	**R/R**	**Low**	**Medium**	**High**	**Low**	**Medium + high**
**Cases ≥5 NMSC and matched controls**											
**Blue**											
Total, n=77	25 (32)	22 (29)	18 (23)	2 (3)	6 (8)	4 (5)	47 (61)	26 (34)	4 (5)	47 (61)	30 (39)
Cases ≥5, n=50	11 (22)	14 (28)	14 (28)	2 (4)	6 (12)	3 (6)	25 (50)	22 (44)	3 (6)	25 (50)	25 (50)
Controls, n=27	14 (52)	8 (29)	4 (15)	0	0	1 (4)	22 (81)	4 (15)	1 (4)	22 (81)	5 (19)
***p* value**	0.059						**0.024**			**0.007**	
**Grey**											
Total, n=27	8 (30)	9 (33)	6 (22)	3 (11)	1 (4)	0	17 (63)	10 (37)	0	17 (63)	10 (37)
Cases ≥5, n=12	3 (25)	4 (33)	4 (33)	0	1 (8)	0	7 (58)	5 (42)	0	7 (58)	5 (42)
Controls, n=15	5 (33)	5 (33)	2 (13)	3 (20)	0	0	10 (67)	5 (33)	0	10 (67)	5 (33)
***p* value**	0.287						0.656			0.656	
**Green**											
Total, n=20	8 (40)	3 (15)	4 (20)	1 (5)	2 (10)	2 (10)	11 (55)	7 (35)	2 (10)	11 (55)	9 (45)
Cases ≥5, n=11	5 (46)	1 (9)	2 (18)	1 (9)	1 (9)	1 (9)	6 (55)	4 (36)	1 (9)	6 (55)	5 (45)
Controls, n=9	3 (34)	2 (22)	2 (22)	0	1 (11)	1 (11)	5 (56)	3 (33)	1 (11)	5 (56)	4 (44)
***p* value**	0.895						0.983			0.964	
**Brown**											
Total, n=50	14 (28)	16 (32)	10 (20)	3 (6)	4 (8)	3 (6)	30 (60)	17 (34)	3 (6)	30 (60)	20 (40)
Cases ≥5, n=14	3 (21)	3 (21)	3 (21)	2 (15)	1 (7)	2 (15)	6 (43)	6 (43)	2 (14)	6 (43)	8 (57)
Controls, n=36	11 (31)	13 (36)	7 (19)	1 (3)	3 (8)	1 (3)	24 (67)	11 (30)	1 (3)	24 (67)	12 (33)
***p* value**	0.362						0.163			0.123	
**Cases ≥10 NMSC and matched controls**											
**Blue**											
Total, n=41	11 (27)	11 (27)	12 (29)	2 (5)	4 (10)	1 (2)	22 (54)	18 (44)	1 (2)	22 (54)	19 (46)
Cases ≥10, n=25	3 (12)	7 (28)	8 (32)	2 (8)	4 (16)	1 (4)	10 (40)	14 (56)	1 (4)	10 (40)	15 (60)
Controls, n=16	8 (50)	4 (25)	4 (25)	0	0	0	12 (75)	4 (25)	0	12 (75)	4 (25)
***p* value**	0.077						0.082			**0.028**	
**Grey**											
Total, n=15	2 (13.3)	8 (53)	3 (20)	2 (13)	0	0	10 (67)	5 (33)	0	10 (67)	5 (33)
Cases ≥10, n=8	1 (13)	4 (50)	3 (38)	0	0	0	5 (63)	3 (37	0	5 (63)	3 (37
Controls, n=7	1 (14)	4 (57)	0	2 (29)	0	0	5 (71)	2 (29)	0	5 (71)	2 (29)
***p* value**	0.175						0.714			0.714	
**Green**											

Total, n=8	2 (25)	2 (25)	1 (13)	1 (13)	1 (13)	1 (13)	4 (50)	3 (38)	1 (13)	4 (50)	4 (50)
Cases ≥10, n=3	1 (33)	0	0	1 (33)	0	1 (33)	1 (33)	1 (33)	1 (33)	1 (33.3)	2 (67)
Controls, n=5	1 (20)	2 (40)	1 (20)	0	1 (20)	0	3 (60)	2 (40)	0	3 (60)	2 (40)
***p* value**	0.319						0.376			0.465	
**Brown**											
Total, n=24	5 (21)	6 (25)	8 (33)	2 (8)	2 (8)	1 (4)	11 (46)	12 (50)	1 (4)	11 (46)	13 (54)
Cases ≥10, n=8	1 (13)	1 (13)	2 (25)	2 (25)	1 (13)	1 (13)	2 (25)	5 (63)	1 (13)	2 (25)	6 (75)
Controls, n=16	4 (25)	5 (31)	6 (38)	0	1 (6)	0	9 (56)	7 (44)	0	9 (56)	7 (44)
***p* value**	0.177						0.173			0.148	
**Cases ≥5 NMSC and cases 1-4 NMSC**											
**Blue**											
Total, n=92	23 (25)	29 (32)	25 (27)	3 (3)	9 (10)	3 (3)	52 (57)	37 (40)	3 (3)	52 (57)	40 (43)
Cases ≥5, n=50	11 (22)	14 (28)	14 (28)	2 (4)	6 (12)	3 (6)	25 (50)	22 (44)	3 (6)	25 (50)	25 (50)
Cases 1-4, n=42	12 (29)	15 (36)	11 (26)	1 (2)	3 (7)	0	27 (64)	15 (36)	0	27 (64)	15 (36)
***p* value**	0.534						0.155			0.169	
**Grey**											
Total, n=24	7 (29)	10 (42)	5 (21)	1 (4)	1 (4)	0	17 (71)	7 (29)	0	17 (71)	7 (29)
Cases ≥5, n=12	3 (25)	4 (33)	4 (33)	0	1 (8)	0	7 (58)	5 (42)	0	7 (58)	5 (42)
Cases 1-4, n=12	4 (33.3)	6 (50)	1 (8)	1 (8)	0	0	10 (83)	2 (17)	0	10 (83)	2 (17)
***p* value**	0.362						0.178			0.178	
**Green**											
Total, n=32	16 (50)	6 (19)	5 (16)	2 (6)	2 (6)	1 (3)	22 (69)	9 (28)	1 (3)	22 (69)	10 (31)
Cases ≥5, n=11	5 (46)	1 (9)	2 (18)	1 (9)	1 (9)	1 (9)	6 (55)	4 (36)	1 (9)	6 (55)	5 (45)
Cases 1-4, n=21	11 (52)	5 (24)	3 (14)	1 (5)	1 (5)	0	16 (76)	5 (24)	0	16 (76)	5 (24)
***p* value**	0.651						0.246			0.210	
**Brown**											
Total, n=46	14 (30)	13 (28)	7 (15)	6 (13)	3 (7)	3 (7)	27 (59)	16 (35)	3 (7)	27 (59)	19 (41)
Cases ≥5, n=14	3 (21)	3 (21)	3 (21)	2 (15)	1 (7)	2 (15)	6 (43)	6 (43)	2 (14)	6 (43)	8 (57)
Cases 1-4, n=32	11 (34)	10 (31)	4 (13)	4 (13)	2 (6)	1 (3)	21 (66)	10 (31)	1 (3)	21 (66)	11 (34)
***p* value**	0.658						0.213			0.149	
**Cases ≥10 NMSC and cases 1-9 NMSC**											
**Blue**											
Total, n=92	23 (25)	29 (32)	25 (27)	3 (3)	9 (10)	3 (3)	52 (57)	37 (40)	3 (3)	52 (57)	40 (43)
Cases ≥10, n=25	3 (12)	7 (28)	8 (32)	2 (8)	4 (16)	1 (4)	10 (40)	14 (56)	1 (4)	10 (40)	15 (60)
Cases 1-9, n=67	20 (30)	22 (33)	17 (25)	1 (2)	5 (8)	2 (3)	42 (63)	23 (34)	2 (3)	42 (63)	25 (37)
***p* value**	0.258						0.146			0.051	
**Grey**											
Total, n=24	7 (29)	10 (42)	5 (21)	1 (4)	1 (4)	0	17 (71)	7 (29)	0	17 (71)	7 (29)
Cases ≥10, n=8	1 (13)	4 (50)	3 (38)	0	0	0	5 (63)	3 (38)	0	5 (63)	3 (38)

Cases 1-9, n=16	6 (38)	6 (38)	2 (13)	1 (6)	1 (6)	0	12 (75)	4 (25)	0	12 (75)	4 (25)
***p* value**	0.414						0.525			0.525	
**Green**											
Total, n=32	16 (50)	6 (19)	5 (16)	2 (6)	2 (6)	1 (3)	22 (69)	9 (28)	1 (3)	22 (69)	10 (31)
Cases ≥10, n=3	1 (33)	0	0	1 (33)	0	1 (33)	1 (33)	1 (33)	1 (33)	1 (33)	2 (67)
Cases 1-9, n=29	15 (52)	6 (21)	5 (17)	1 (3)	2 (7)	0	21 (72)	8 (28)	0	21 (72)	8 (28)
***p* value**	**0.010**						**0.006**			**0.040**	
**Brown**											
Total, n=46	14 (31)	13 (28)	7 (15)	6 (13)	3 (7)	3 (7)	27 (59)	16 (34)	3 (7)	27 (59)	19 (41)
Cases ≥10, n=8	1 (13)	1 (13)	2 (25)	2 (25)	1 (13)	1 (13)	2 (25)	5 (63)	1 (13)	2 (25)	6 (75)
Cases 1-9, n=38	13 (34)	12 (32)	5 (13)	4 (11)	2 (5)	2 (5)	25 (66)	11 (29)	2 (5)	25 (66)	13 (34)
***p* value**	0.466						0.103			**0.033**	

p values were calculated using the chi-square test, $p<0.05$ is considered significant, indicated in bold.

Table S24a, part I. Association of *MC1R* risk groups with different UV scenarios in cases versus controls

		MC1R						*MC1R*		
		**0/0**	**0/r**	**0/R**	**R/r**	**r/r**	**R/R**	**Low**	**Medium**	**High**
		Total: n=131<br>Cases: n=60<br>Controls: n=71	Total: n=126<br>Cases: n=58<br>Controls: n=68	Total: n=69<br>Cases: n=42<br>Controls: n=27	Total: n=26<br>Cases: n=12<br>Controls: n=14	Total: n=24<br>Cases: n=15<br>Controls: n=9	Total: n=12<br>Cases: n=7<br>Controls: n=5	Total: n=257<br>Cases: n=118<br>Controls: n=139	Total: n=119<br>Cases: n=69<br>Controls: n=50	Total: n=12<br>Cases: n=7<br>Controls: n=5
**Time spent outside with unintentional sun exposure** *(May-August)*										
**Outdoor occupation** Total hours *(mean ± SD)*	Total	5471 ± 9314	4571 ± 8711	6614 ± 10603	1822 ± 4176	3467 ± 5852	3740 ± 7834	5030 ± 9016	4932 ± 8909	3740 ± 7834
	Cases	5645 ± 9777	3110 ± 6409	6061 ± 10362	2800 ± 5864	2773 ± 5878	4697 ± 9870	4399 ± 8356	4779 ± 8944	4697 ± 9870
	Controls	5324 ± 8971	5818 ± 10159	7476 ± 11111	983 ± 1673	4622 ± 5965	2400 ± 4336	5565 ± 9538	5144 ± 8947	2400 ± 4336
	***p* value**	0.845	0.082	0.592	0.277	0.466	0.639	0.302	0.827	0.639
**Total time spent outside with unintentional sun exposure** (including occupation; **UV-scenario I+II**) Total hours *(mean ± SD)*	Total	29159 ± 15854	28187 ± 15698	29180 ± 14626	21748 ± 7487	28444 ± 17100	25575 ± 11358	28682 ± 15754	27408 ± 14186	25575 ± 11358
	Cases	29376 ± 12608	27512 ± 17412	27699 ± 12136	20943 ± 9618	28670 ± 20847	28889 ± 8554	28459 ± 15123	26731 ± 14164	28889 ± 8554
	Controls	28976 ± 18241	28763 ± 14182	31486 ± 17836	22438 ± 5329	28068 ± 8935	20934 ± 14102	28871 ± 16322	28337 ± 14310	20934 ± 14102
	***p* value**	0.886	0.658	0.297	0.622	0.936	0.249	0.835	0.545	0.249
**Time spent outside with intentional sun exposure** *(9am-3pm, May-August)*										
**Central Europe (UV-scenario III)** Total hours *(mean ± SD)*	Total	5691 ± 4030	5352 ± 4075	6456 ± 4203	4954 ± 2436	5342 ± 3155	6728 ± 4773	5524 ± 4048	5903 ± 3717	6728 ± 4773
	Cases	6075 ± 3728	5431 ± 3440	7086 ± 3748	4629 ± 2477	5462 ± 3796	7695 ± 5585	5758 ± 3588	6305 ± 3668	7695 ± 5585
	Controls	5366 ± 4268	5285 ± 4573	5476 ± 4734	5232 ± 2457	5143 ± 1824	5374 ± 3453	5326 ± 4403	5347 ± 3749	5374 ± 3453
	***p* value**	0.318	0.841	0.121	0.539	0.816	0.432	0.394	0.166	0.432
**Southern geographic regions (UV-scenario IV)** Total hours *(mean ± SD)*	Total	2632 ± 2467	2995 ± 2874	2801 ± 2727	2293 ± 1630	2567 ± 2299	2350 ± 2431	2809 ± 2675	2642 ± 2433	2350 ± 2431
	Cases	2870 ± 2587	3089 ± 2493	2952 ± 2942	2274 ± 1207	2804 ± 2394	2647 ± 3024	2977 ± 2532	2801 ± 2588	2647 ± 3024
	Controls	2431 ± 2361	2914 ± 3179	2567 ± 2388	2310 ± 1968	2171 ± 2210	1934 ± 1471	2667 ± 2791	2423 ± 2208	1934 ± 1471
	***p* value**	0.313	0.735	0.571	0.956	0.526	0.640	0.356	0.405	0.640
**Total time spent outside with intentional sun exposure (UV-scenario III+IV)** Total hours *(mean ± SD)*	Total	8323 ± 4373	8347 ± 5022	9258 ± 5636	7247 ± 2871	7909 ± 4133	9078 ± 6054	8334 ± 4693	8546 ± 4912	9078 ± 6054
	Cases	8945 ± 4030	8521 ± 4821	10038 ± 5647	6902 ± 2686	8266 ± 5114	10341 ± 7278	8736 ± 4422	9107 ± 5227	10341 ± 7278
	Controls	7798 ± 4606	8199 ± 5217	8043 ± 5502	7542 ± 3089	7314 ± 1645	7308 ± 3827	7993 ± 4900	7771 ± 4373	7308 ± 3827
	***p* value**	0.135	0.721	0.153	0.581	0.596	0.418	0.207	0.144	0.418

**Outdoor leisure time activities during vacation**										
**Body covered** (mountaineering, hiking, skiing) Total weeks *(mean ± SD)*	Total	57 ± 88	72 ± 125	54 ± 80	101 ± 203	62 ± 64	54 ± 64	64 ± 107	65 ± 116	54 ± 64
	Cases	61 ± 80	70 ± 113	62 ± 92	76 ± 114	56 ± 47	62 ± 72	65 ± 97	62 ±87	62 ± 72
	Controls	53 ± 94	73 ± 135	42 ± 55	123 ± 259	72 ± 88	42 ± 58	63 ± 116	70 ± 148	42 ± 58
	***p* value**	0.601	0.889	0.313	0.563	0.563	0.633	0.846	0.744	0.633
**Wearing swimwear** (water sports, sunbathing) Total weeks *(mean ± SD)*	Total	40 ± 71	39 ± 68	50 ± 74	44 ± 58	55 ± 91	12 ± 21	40 ± 69	50 ± 74	12 ± 21
	Cases	56 ± 93	27 ± 41	44 ± 60	48 ± 65	42 ± 83	20 ± 25	42 ± 73	44 ± 65	20 ± 25
	Controls	27 ± 41	49 ± 83	60 ± 92	41 ± 54	77 ± 104	0	38 ± 65	58 ± 84	0
	***p* value**	**0.020**	0.070	0.391	0.771	0.371	0.104	0.641	0.331	0.104
**Full body uncovered** (nudist beach) Total weeks *(mean ± SD)*	Total	3 ± 13	3 ± 13	2 ± 7	6 ± 17	12 ± 28	20 ± 69	5 ± 14	4 ± 16	20 ± 69
	Cases	3 ± 13	5 ± 18	1 ± 6	6 ± 17	11 ± 31	34 ± 91	7 ± 15	4 ± 17	34 ± 91
	Controls	2 ± 12	1 ± 5	2 ± 8	6 ± 18	13 ± 24	0	4 ± 12	4 ± 15	0
	***p* value**	0.713	0.089	0.539	0.956	0.863	0.422	0.136	0.745	0.422
**Sunbed use** *(sun-bed users only; total n=20)* Total hours *(mean ± SD)*	Total	35 ± 10	36 ± 14	34 ± 9	34 ± 13	28 ± 9	35 ± 10	35 ± 12	33 ± 9	35 ± 10
	Cases	35 ± 11	35 ± 15	31 ± 8	30 ± 13	24 ± 5	45 ± 0	35 ± 13	29 ± 9	45 ± 0
	Controls	34 ± 10	36 ± 12	39 ± 9	46 ± 0	40 ± 0	30 ± 7	35 ± 11	40 ± 7	30 ± 7
	***p* value**	0.699	0.473	0.152	0.823	0.173	0.422	0.928	**0.015**	0.333
**Gardening** *(gardeners only; total n=92)* Total hours *(mean ± SD)*	Total	4059± 4091 92	4864 ± 7156 89	3420 ± 4937	3176 ± 3126	2858 ± 2417	4360 ± 6594	4454 ± 5802 181	3907 ± 4420 ?	4360 ± 6594 ?
	Cases	3765 ± 3860 39	3935 ± 4373 40	3700 ± 5417	4623 ± 3881	2827 ± 2330	6469 ± 8015	3851 ± 4102 79	4357 ± 4884 ?	6469 ± 8015 ?
	Controls	4275± 4277 53	5622 ± 8778 49	2984 ± 4140	1874 ± 1477	2929 ± 2812	1408 ± 2130	4922 ± 6812 102	3244 ± 3594 ?	1408 ± 2130 ?
	***p* value**	0.138	0.271	0.560	0.053	0.941	0.204	0.219	0.246	0.204

$p<0.05$ is considered significant, indicated in bold.

Table S24a, part II. Association of *MC1R* risk groups with different UV scenarios in cases versus controls

	MC1R	
	**Low**	**Medium +high**
	Total: n=257<br>Cases: n=118<br>Controls: n=139	Total: n=119<br>Cases: n=69<br>Controls: n=50
**Time spent outside with unintentional sun exposure** *(May-August)*		

**Outdoor occupation**	Total	5030 ± 9016	4787 ± 8772
Total hours *(mean ± SD)*	Cases	4399 ± 8356	4772 ± 8963
	Controls	5565 ± 9538	4807 ± 8587
	***p* value**	0.302	0.982
**Total time spent outside with unintentional sun exposure** (including occupation; **UV- scenario I+II**)	Total	28682 ± 15754	27183 ± 13886
Total hours *(mean ± SD)*	Cases	28459 ± 15123	26933 ± 13715
	Controls	28871 ± 16322	27522 ± 14233
	***p* value**	0.835	0.811
**Time spent outside with intentional sun exposure** *(9am-3pm, May-August)*			
**Central Europe (UV-scenario III)**	Total	5524 ± 4048	5933 ± 3832
Total hours *(mean ± SD)*	Cases	5758 ± 3588	6434 ± 3855
	Controls	5326 ± 4403	5255 ± 3728
	***p* value**	0.394	0.081
**Southern geographic regions (UV-scenario IV)**	Total	2809 ± 2675	2624 ± 2417
Total hours *(mean ± SD)*	Cases	2977 ± 2532	2788 ± 2609
	Controls	2667 ± 2791	2401 ± 2133
	***p* value**	0.356	0.366
**Total time spent outside with intentional sun exposure (UV-scenario III+IV)**	Total	8334 ± 4693	8557 ± 5003
Total hours *(mean ± SD)*	Cases	8736 ± 4422	9221 ± 5399
	Controls	7993 ± 4900	7656 ± 4293
	***p* value**	0.207	0.076
**Outdoor leisure time activities during vacation**			
**Body covered** (mountaineering, hiking, skiing)	Total	64 ± 107	65 ± 112
Total weeks *(mean ± SD)*	Cases	65 ± 97	63 ± 86
	Controls	63 ± 116	67 ± 141
	***p* value**	0.846	0.831
**Wearing swimwear** (water sports, sunbathing)	Total	40 ± 69	46 ± 71
Total weeks *(mean ± SD)*	Cases	42 ± 73	42 ± 63
	Controls	38 ± 65	52 ± 81
	***p* value**	0.641	0.421
**Full body uncovered** (nudist beach)	Total	5 ± 14	6 ± 26
Total weeks *(mean ± SD)*	Cases	7 ± 15	7 ± 32
		4 ± 12	5 ± 14

	Controls	0.136	0.604
	***p* value**		
**Sunbed use** (*sun-bed users only; total n=20*) Total hours (*mean ± SD*)	Total	35 ± 12	34 ± 11
	Cases	35 ± 13	33 ± 10
	Controls	35 ± 11	35 ± 9
	***p* value**	0.928	0.661
**Gardening** (*gardeners only; total n=92*) Total hours (*mean ± SD*)	Total	4454 ± 5802	4124 ± 4715
	Cases	3851 ± 4102	4682 ± 5305
	Controls	4922 ± 6812	3259 ± 3510
	***p* value**	0.219	0.148

p<0.05 is considered significant, indicated in bold.

Table S24b, part I. Association of *MC1R* risk groups with different UV scenarios in cases with ≥ 10 NMSC (n=44) versus matched controls

		MC1R						*MC1R*	
		**0/0**	**0/r**	**0/R**	**R/r**	**r/r**	**R/R**	**Low**	**Medium + high**
		Total: n=21 Cases: n=6 Controls: n=15	Total: n=26 Cases: n=12 Controls: n=14	Total: n=24 Cases: n=13 Controls: n=11	Total: n=7 Cases: n=5 Controls: n=2	Total: n=7 Cases: n=5 Controls: n=2	Total: n=3 Cases: n=3 Controls: n=0	Total: n=47 Cases: n=18 Controls: n=29	Total: n=41 Cases: n=26 Controls: n=15
**Time spent outside with unintentional sun exposure** (*May-August*) **Outdoor occupation** Total hours (*mean ± SD*)	Total	4586 ± 7917	4860 ± 9446	4173 ± 8015	2491 ± 3403	5257 ± 6957	8800 ± 15242	4739 ± 8705	4410 ± 7733
	Cases ≥10	5333 ± 13064	1867 ± 3114	1797 ± 2902	2880 ± 3944	4160 ± 5943	8800 ± 15242	3022 ± 7701	3268 ± 5980
	controls	4288 ± 5317	7428 ± 12126	6982 ± 11028	1520 ± 2150	8000 ± 11314	-	5804 ± 9242	6389 ± 10019
	***p* value**	0.792	0.137	0.116	0.675	0.559	n.a.	0.292	0.217
**Total time spent outside with unintentional sun exposure** (including occupation; **UV- scenario I+II**) Total hours (*mean ± SD*)	Total	28616 ± 13829	30511 ± 20323	27173 ± 13359	23890 ± 7086	26896 ± 11515	31029 ± 5787	29700 ± 17569	26847 ± 11603
	Cases ≥10	30008 ± 20341	30987± 24277	24499 ± 8794	26422 ± 6374	26640 ± 13959	31029 ± 5787	30660 ± 22434	26034 ± 9044
	controls	28059 ± 11148	30221 ± 17172	30333 ± 17246	17560 ± 5148	27536 ± 3869	-	29103 ± 14164	28257 ± 15336
	***p* value**	0.779	0.926	0.297	0.146	0.936	n.a.	0.771	0.561
**Time spent outside with intentional sun exposure** (*9am-3pm, May-August*) **Central Europe (UV-scenario III)** Total hours (*mean ± SD*	Total	5964 ± 5038	6430 ± 3898	7230 ± 4625	5375 ± 2570	8414 ± 2887	10060 ± 8783	6222 ± 4399	7323 ± 4454
	Cases ≥10	3881 ± 2966	6674 ± 3319	8290 ± 3941	5543 ± 2283	9089 ± 3236	10060 ± 8783	5743 ± 3399	8119 ± 4256
	Controls	6798 ± 5523	6221 ± 4454	5978 ± 5232	4956 ± 4277	6729 ± 420	-	6520 ± 4954	5942 ± 4594
	***p* value**	0.240	0.775	0.230	0.812	0.375	n.a.	0.562	0.133

**Southern geographic regions (UV-scenario IV)**	Total	2604 ± 2332	2633 ± 2609	3443 ± 3313	2094 ± 1513	2505 ± 2911	2606 ± 4226	2620 ± 2463	2991 ± 3022
	Cases ≥10	3469 ± 2738	3769 ± 3224	4817 ± 3794	2453 ± 1671	3154 ± 3256	2606 ± 4226	3669 ± 2992	3787 ±3411
Total hours *(mean ± SD)*	Controls	2258 ± 2155	1659 ± 1447	1819 ± 1616	1197 ± 564	882 ± 1035	-	1970 ± 1841	1611 ± 1448
	***p* value**	**0.294**	**0.037**	**0.023**	0.368	0.399	n.a.	**0.020**	**0.024**
**Total time spent outside with intentional sun exposure (UV-scenario III+IV)**	Total	8569 ± 5218	9063 ± 5203	10673 ± 6639	7469 ± 2710	10919 ± 5118	12666 ±11422	8843 ± 5159	10313 ± 6218
	Cases ≥10	7350 ± 3210	10442 ± 5309	13106 ± 6760	7996 ± 2522	12242 ± 5616	12666 ±11422	9411 ± 4850	11907 ± 6481
	Controls	9057 ± 5859	7881 ± 4995	7797 ± 5452	6153 ± 3712	7611 ± 615	-	8490 ± 5395	7553 ± 4751
	***p* value**	0.512	0.218	**0.048**	0.467	0.321	n.a.	0.557	**0.029**
**Outdoor leisure time activities during vacation**									
**Body covered** (mountaineering, hiking, skiing)	Total	59 ± 94	78 ± 89	59 ± 93	47 ± 40	42 ± 29	71 ± 61	70 ± 91	55 ± 74
	Cases ≥10	27 ± 30	77 ± 84	70 ± 110	57 ± 44	43 ± 33	71 ± 61	60 ± 73	62 ± 82
	Controls	72 ± 109	80 ± 94	46 ± 70	23 ± 11	41 ± 28	-	76 ± 100	42 ± 60
Total weeks *(mean ± SD)*	***p* value**	0.337	0.936	0.535	0.354	0.929	n.a.	0.571	0.404
**Wearing swimwear**	Total	29 ± 43	33 ± 57	53 ± 82	35 ± 66	51 ± 123	3 ± 6	31 ± 51	46 ± 83
(water sports, sunbathing)	Cases ≥10	49 ± 50	19 ± 20	53 ± 65	41 ± 78	72 ± 145	3 ± 6	29 ± 34	49 ± 82
Total weeks *(mean ± SD)*	Controls	21 ± 39	43 ± 75	54 ± 102	20 ± 28	0 ± 0	-	32 ± 59	42 ± 89
	***p* value**	0.186	0.285	0.996	0.738	0.536	n.a.	0.858	0.803
**Full body uncovered**	Total	2 ± 9	5 ± 23	2 ± 8	9 ± 22	9 ± 15	0 ± 1	4 ±18	4 ± 12
(nudist beach)	Cases ≥10	0	10 ± 35	4 ± 11	13 ± 26	6 ± 13	0 ± 1	7 ± 28	5 ± 15
Total weeks *(mean ± SD)*	Controls	3 ± 10	0 ± 1	1 ± 3	0 ± 1	15 ± 21	-	2 ± 7	3 ± 8
	***p* value**	0.472	0.303	0.429	0.532	0.513	n.a.	0.378	0.486
**Sunbed use** (*sun-bed users only; total n=)*	Total	32 ± 7	32.4 ± 16	32 ± 6	25 ± 0	-	-	32 ±12	30 ± 6
	Cases ≥10	35 ± 7	16 ± 15	31 ± 7	25 ± 0	-	-	29 ± 12	30 ± 7
Total hours *(mean ± SD)*	Controls	25 ± 7	37 ± 14	35 ± 0	-	-	-	34 ± 14	35 ± 0
	***p* value**	0.454	0.110	0.625	n.a.	n.a.	n.a.	0.583	0.495
**Gardening** (*gardeners only; total n=)*	Total	5001 ± 6130	7047 ± 10229	2651 ±3590	5109 ±4221	2903 ± 3318	11360 ± 10961	4208 ± 7458	3751 ± 4822
Total hours *(mean ± SD)*	Cases ≥10	5168 ± 5887	2491 ± 1269	2775 ± 3990	5680 ± 4967	1568 ± 2772	11360 ± 10961	2117 ± 3297	4092 ± 5608
	Controls	4940 ± 6436	2590 ± 1274	2505 ± 3238	3680 ± 1584	6240 ± 2036	-	5506 ± 8957	3159 ± 3114
	***p* value**	0.953	0.919	0.859	0.681	0.088	n.a.		

P<0.05 is considered significant, indicated in bold; n.a.=not applicable.

Table S24b, part II. Association of *MC1R* risk groups with different UV scenarios in cases with ≥ 10 NMSC versus controls

		MC1R			*MC1R*	
		**Low**<br>Total: n=47<br>Cases: n=18<br>Controls: n=29	**Medium**<br>Total: n=38<br>Cases: n=23<br>Controls: n=15	**High**<br>Total: n=3<br>Cases: n=3<br>Controls: n=0	**Low**<br>Total: n=47<br>Cases: n=18<br>Controls: n=29	**Medium + high**<br>Total: n=41<br>Cases: n=26<br>Controls: n=15
**Time spent outside with unintentional sun exposure** *(May-August)*						
**Outdoor occupation**	Total	4739 ± 8705	3939 ± 8605	8800 ± 15242	4739 ± 8705	4410 ± 7733
Total hours *(mean ± SD)*	Cases ≥10	3022 ± 7701	3122 ± 7531	8800 ± 15242	3022 ± 7701	3268 ± 5980
	Controls	5804 ± 9242	5924 ± 9167	-	5804 ± 9242	6389 ± 10019
	***p* value**	0.292	0.356	n.a.	0.292	0.217
**Total time spent outside with unintentional sun exposure** (including occupation; **UV- scenario I+II**)	Total	29700 ± 17569	30841 ± 17569	31029 ± 5787	29700 ± 17569	26847 ± 11603
Total hours *(mean ± SD)*	Cases ≥10	30660 ± 22434	30430 ± 22434	31029 ± 5787	30660 ± 22434	26034 ± 9044
	Controls	29103 ± 14164	29900 ± 14164	-	29103 ± 14164	28257 ± 15336
	***p* value**	0.771	0.811	n.a.	0.771	0.561
**Time spent outside with intentional sun exposure** *(9am-3pm, May-August)*	Total	6222 ± 4399	6655 ± 4399	10060 ± 8783	6222 ± 4399	7323 ± 4454
**Central Europe (UV-scenario III)**	Cases ≥10	5743 ± 3399	5911 ± 3459	10060 ± 8783	5743 ± 3399	8119 ± 4256
Total hours *(mean ± SD*	Controls	6520 ± 4954	6113 ± 4953	-	6520 ± 4954	5942 ± 4594
	***p* value**	0.562	0.572	n.a.	0.562	0.133
**Southern geographic regions (UV-scenario IV)**	Total	2620 ± 2463	28i0 ± 2422	2606 ± 4226	2620 ± 2463	2991 ± 3022
Total hours *(mean ± SD)*	Cases ≥10	3669 ± 2992	3669 ± 2992	2606 ± 4226	3669 ± 2992	3787 ±3411
	Controls	1970 ± 1841	2370 ± 1242	-	1970 ± 1841	1611 ± 1448
	***p* value**	**0.020**	0.071	n.a.	**0.020**	**0.024**
**Total time spent outside with intentional sun exposure (UV-scenario III+IV)**	Total	8843 ± 5159	8733 ± 5088	12666 ±11422	8843 ± 5159	10313 ± 6218
	Cases ≥10	9411 ± 4850	9222 ± 4710	12666 ±11422	9411 ± 4850	11907 ± 6481
	Controls	8490 ± 5395	8101 ± 5235	-	8490 ± 5395	7553 ± 4751
	***p* value**	0.557	0.643	n.a.	0.557	**0.029**
**Outdoor leisure time activities during vacation**	Total	70 ± 91	80 ± 90	71 ± 61	70 ± 91	55 ± 74
**Body covered**	Cases ≥10	60 ± 73	79 ± 71	71 ± 61	60 ± 73	62 ± 82
(mountaineering, hiking, skiing)	Controls	76 ± 100	76 ± 103	-	76 ± 100	42 ± 60
Total weeks *(mean ± SD)*	***p* value**	0.571	0.843	n.a.	0.571	0.404
**Wearing swimwear**	Total	31 ± 51	35 ± 56	3 ± 6	31 ± 51	46 ± 83
(water sports, sunbathing)	Cases ≥10	29 ± 34	30 ± 33	3 ± 6	29 ± 34	49 ± 82
Total weeks *(mean ± SD)*	Controls	32 ± 59	31 ± 49	-	32 ± 59	42 ± 89

	***p* value**	0.858	0.888	n.a.	0.858	0.803
**Full body uncovered** (nudist beach) Total weeks *(mean ± SD)*	Total	4 ±18	4 ±12	0 ± 1	4 ±18	4 ± 12
	Cases ≥10	7 ± 28	6 ± 26	0 ± 1	7 ± 28	5 ± 15
	Controls	2 ± 7	3 ± 5	-	2 ± 7	3 ± 8
	***p* value**	0.378	0.255	n.a.	0.378	0.486
**Sunbed use** *(sun-bed users only)* Total hours *(mean ± SD)*	Total	32 ±12	34 ±14	-	32 ±12	30 ± 6
	Cases ≥10	29 ± 12	34 ± 14	-	29 ± 12	30 ± 7
	Controls	34 ± 14	36 ± 16	-	34 ± 14	35 ± 0
	***p* value**	0.583	0.511	n.a.	0.583	0.495
**Gardening** (*gardeners only)* Total hours *(mean ± SD)*	Total	4208 ± 7458	4119 ± 7321	11360 ± 10961	4208 ± 7458	3751 ± 4822
	Cases ≥10	2117 ± 3297	2011 ± 2981	11360 ± 10961	2117 ± 3297	4092 ± 5608
	Controls	5506 ± 8957	5120 ± 9057	-	5506 ± 8957	3159 ± 3114
	***p* value**	0.131	0.168	n.a.	0.131	0.557

p<0.05 is considered significant, indicated in bold; n.a.=not applicable.

Table S25. Association of *MC1R* risk groups with hair color in different subgroups of OTR

	MC1R						*MC1R*			*MC1R*	
	**0/0**	**0/r**	**0/R**	**R/r**	**r/r**	**R/R**	**Low**	**Medium**	**High**	**Low**	**Medium + high**
**Cases ≥5 NMSC and cases 1-4 NMSC**											
**Red**											
Total, n=2	0	0	0	0	0	2 (100)	0	0	2 (100)	0	2 (100)
Cases ≥5, n=2	0	0	0	0	0	2 (100)	0	0	2 (100)	0	2 (100)
Cases 1-4, n=0	0	0	0	0	0	0	0	0	0	0	0
***p* value**	-						-			-	
**Blond**											
Total, n=74	22 (30)	21 (28)	18 (24)	2 (3)	7 (10)	4 (5)	43 (58)	27 (37)	4 (5)	43 (58)	31 (42)
Cases ≥5, n=34	10 (29)	5 (15)	9 (26)	1 (3)	5 (15)	4 (12)	15 (44)	15 (44)	4 (12)	15 (44)	19 (56)
Cases 1-4, n=40	12 (30)	16 (40)	9 (23)	1 (3)	2 (5)	0	28 (70)	12 (30)	0	28 (70)	12 (30)
***p* value**	0.055						**0.020**			**0.025**	
**Light brown**											
Total, n=60	14 (23)	18 (30)	16 (27)	6 (10)	5 (8)	1 (2)	32 (53)	27 (45)	1 (2)	32 (53)	28 (47)
Cases ≥5, n=24	6 (25)	6 (25)	7 (29)	2 (8)	3 (13)	0	12 (50)	12 (50)	0	12 (50)	12 (50)
Cases 1-4, n=36	8 (22)	12 (33)	9 (25)	4 (11)	2 (6)	1 (3)	20 (55)	15 (42)	1 (3)	20 (55)	16 (44)
***p* value**	0.837						0.615			0.673	
**Dark brown**											
Total, n=52	21 (40)	17 (33)	7 (13)	4 (8)	3 (6)	0	38 (73)	14 (27)	0	38 (73)	14 (27)
Cases ≥5, n=25	6 (24)	10 (40)	6 (24)	2 (8)	1 (4)	0	16 (64)	9 (36)	0	16 (64)	9 (36)
Cases 1-4, n=27	15 (56)	7 (26)	1 (4)	2 (7)	2 (7)	0	22 (82)	5 (19)	0	22 (82)	5 (19)
***p* value**	0.084						0.156			0.156	
**Black**											
Total, n=6	3 (50)	2 (33)	1 (17)	0	0	0	5 (83)	1 (17)	0	5 (83)	1 (17)
Cases ≥5, n=2	0	1 (50)	1 (50)	0	0	0	1 (50)	1 (50)	0	1 (50)	1 (50)
Cases 1-4, n=4	3 (75)	1 (25)	0	0	0	0	4 (100)	0	0	4 (100)	0
***p* value**	0.153						0.121			0.121	
**Cases ≥10 NMSC and cases 1-9 NMSC**											
**Red**											
Total, n=2	0	0	0	0	0	2 (100)	0	0	2 (100)	0	2 (100)
Cases ≥10, n=1	0	0	0	0	0	1 (100)	0	0	1 (100)	0	1 (100)
Cases 1-9, n=1	0	0	0	0	0	1 (100)	0	0	1 (100)	0	1 (100)
***p* value**	-						-			-	
**Blond**											
Total, n=74	22 (30)	21 (29)	18 (24)	2 (3)	7 (9)	4 (5)	43 (59)	27 (36)	4 (5)	43 (59)	31 (41)
Cases ≥10, n=17	4 (24)	4 (24)	4 (24)	1 (6)	2 (11)	2 (12)	8 (47)	7 (41)	2 (12)	8 (47)	9 (53)
Cases 1-9, n=57	18 (31)	17 (30)	14 (25)	1 (2)	5 (9)	2 (3)	35 (61)	20 (35)	2 (4)	35 (61)	22 (39)
***p* value**	0.688						0.325			0.293	
**Light brown**											
Total, n=60	14 (23)	18 (30)	16 (27)	6 (10)	5 (8)	1 (2)	32 (53)	27 (45)	1 (2)	32 (53)	28 (47)
Cases ≥10, n=12	1 (8)	3 (25)	4 (33)	2 (17)	2 (17)	0	4 (33)	8 (67)	0	4 (33)	8 (67)
Cases 1-9, n=48	13 (27)	15 (32)	12 (25)	4 (8)	3 (6)	1 (2)	28 (58)	19 (40)	1 (2)	28 (58)	20 (42)
***p* value**	0.551						0.230			0.121	
**Dark brown**											
Total, n=52	21 (40)	17 (33)	7 (13)	4 (8)	3 (6)	0	38 (73)	14 (27)	0	38 (73)	14 (27)
Cases ≥10, n=13	1 (7)	4 (31)	5 (39)	2 (15)	1 (8)	0	5 (39)	8 (62)	0	5 (39)	8 (62)

	Cases 1-9, n=39	20 (52)	13 (33)	2 (5)	2 (5)	2 (5)	0	33 (85)	6 (15)	0	33 (85)	6 (15)
	***p* value**	**0.007**						**0.001**			**0.001**	
**Black**												
	Total, n=6	3 (50)	2 (33)	1 (17)	0	0	0	5 (83)	1 (17)	0	5 (83)	1 (17)
	Cases ≥10, n=1	0	1 (100)	0	0	0	0	1 (100)	0	0	1 (100)	0
	Cases 1-9, n=5	3 (60)	1 (20)	1 (20)	0	0	0	4 (80)	1 (20)	0	4 (80)	1 (20)
	***p* value**	0.301						0.624			0.624	

p values were calculated using the chi-square test, $p<0.05$ is considered significant, indicated in bold.

Table S26. Association of *MC1R* risk groups with different NMSC entities

Tumor entity *(n (%))*		*MC1R*						*MC1R*			*MC1R*	
		**0/0**	**0/r**	**0/R**	**R/r**	**r/r**	**R/R**	**Low**	**Medium**	**High**	**Low**	**Medium + high**
**NMSC**	194	60 (31)	58 (30)	42 (22)	12 (6)	15 (8)	7 (4)	118 (61)	69 (36)	7 (4)	118 (61)	76 (39)
**cSCC only**	34 (18)	14 (41)	10 (29)	8 (24)	0	1 (3)	1 (3)	24 (70.5)	9 (27)	1 (3)	24 (71)	10 (30)
**BCC only**	39 (20)	13 (33)	16 (41)	5 (13)	3 (8)	2 (5)	0	29 (74)	10 (26)	0	29 (74)	10 (26)
**BD only**	5 (3)	1 (20)	3 (60)	1 (20)	0	0	0	4 (80)	1 (20)	0	4 (80)	1 (20)
**cSCC + BCC**	28 (14)	7 (25)	7 (25)	5 (18)	3 (11)	6 (21)	0	14 (50)	14 (50)	0	14 (50)	14 (50)
**cSCC + BD**	16 (8)	6 (38)	2 (13)	4 (25)	1 (6)	2 (13)	1 (6)	8 (50)	7 (44)	1 (6)	8 (50)	8 (50)
**BCC + BD**	13 (7)	6 (46)	3 (23)	2 (15)	2 (8)	1 (8)	0	9 (69)	4 (31)	0	9 (69)	4 (31)
**cSCC + BCC + BD**	59 (30)	13 (22)	17 (29)	17 (29)	4 (7)	3 (5)	5 (8)	30 (51)	24 (41)	5 (8)	30 (51)	29 (49)

NMSC=non melanoma skin cancer, cSCC=cutaneous squamous cell carcinoma including Bowen's carcinoma, BCC=basal cell carcinoma, BD=Bowen´s disease.

Figure S1. Hours of high UV-exposure (9am-3pm) in Mediterranean or southern regions in cases versus controls.

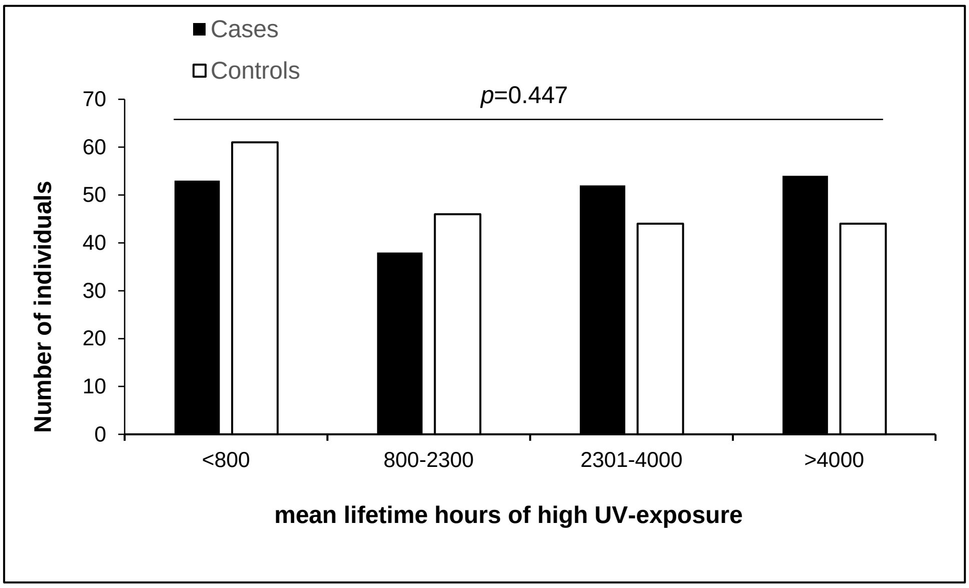

Mean lifetime hours are divided into quartiles. The *p* value was calculated using the chi-square test, $p<0.05$ is considered significant.
